# Targeting tumor immunosuppressive microenvironment for pancreatic cancer immunotherapy: Current research and future perspective

**DOI:** 10.3389/fonc.2023.1166860

**Published:** 2023-03-29

**Authors:** Ying Li, Shuai Xiang, Wenjun Pan, Jing Wang, Hanxiang Zhan, Shanglong Liu

**Affiliations:** ^1^ Department of Blood Transfusion, the Affiliated Hospital of Qingdao University, Qingdao, China; ^2^ Department of Gastrointestinal Surgery, the Affiliated Hospital of Qingdao University, Qingdao, China; ^3^ Department of Operating Room, the Affiliated Hospital of Qingdao University, Qingdao, China; ^4^ Department of General Surgery, Qilu hospital, Shandong University, Jinan, Shandong, China

**Keywords:** pancreatic cancer, immunosuppression, tumor microenvironment, immunotherapy, clinical trial

## Abstract

Pancreatic cancer is one of the most malignant tumors with increased incidence rate. The effect of surgery combined with chemoradiotherapy on survival of patients is unsatisfactory. New treatment strategy such as immunotherapy need to be investigated. The accumulation of desmoplastic stroma, infiltration of immunosuppressive cells including myeloid derived suppressor cells (MDSCs), tumor associated macrophages (TAMs), cancer‐associated fibroblasts (CAFs), and regulatory T cells (Tregs), as well as tumor associated cytokine such as TGF-β, IL-10, IL-35, CCL5 and CXCL12 construct an immunosuppressive microenvironment of pancreatic cancer, which presents challenges for immunotherapy. In this review article, we explore the roles and mechanism of immunosuppressive cells and lymphocytes in establishing an immunosuppressive tumor microenvironment in pancreatic cancer. In addition, immunotherapy strategies for pancreatic cancer based on tumor microenvironment including immune checkpoint inhibitors, targeting extracellular matrix (ECM), interfering with stromal cells or cytokines in TME, cancer vaccines and extracellular vesicles (EVs) are also discussed. It is necessary to identify an approach of immunotherapy in combination with other modalities to produce a synergistic effect with increased response rates in pancreatic cancer therapy.

## Introduction

Pancreatic cancer is one of the most malignant digestive system tumors, ranking the fourth most common cause of cancer death with increased incidence rate (1). Surgical treatment is one of the most effective way, but the rate of radical resection is less than 20%. The main causes of death following operation are the recurrence and metastasis. Moreover, pancreatic cancer is striking resistance to radiotherapy and chemotherapy (2). It is necessary to explore a new therapeutic approach. Therefore, immunotherapy has been given high hopes and now become a key pillar of cancer treatment alongside chemoradiotherapy. In pancreatic cancer, cytotoxic T lymphocytes are suppressed by immunosuppressive interaction between cancer cells with stromal cells in tumor microenvironment (TME) like cancer‐associated fibroblasts (CAFs), myeloid derived suppressor cells (MDSCs), regulatory T cells (Tregs), and tumor associated macrophages (TAMs). Although various immunotherapy methods are different, it is suggested to use a combination of immunotherapeutic approaches for helping active cytotoxic T lymphocytes (CTLs) infiltrate the pancreatic TME or rescuing the exhausted CTLs (3). Therefore, maintaining the activated state of CTLs in TME is the key to tumor immunotherapy. The TME of pancreatic cancer is characterized by low oxygen, acid, high permeability, a large number of growth factors and hydrolases, resulting in insensitivity to apoptosis, resistance to growth inhibition, immune tolerance and immune escape (4). Immune microenvironment remodeling in pancreatic cancer plays an important role in the development of malignant tumors, which poses a challenge to the immunotherapy of pancreatic cancer. First, pancreatic cancer lacks the expression of immunogenic antigens, and their major histocompatibility antigens (MHC) I molecules are significantly downregulated. Secondly, the activities of effector T cells including natural killer (NK) cells, dendritic cells (DCs) and macrophages are significantly decreased, while inhibitory immune cells including Tregs, MDSCs and M2-TAMs aggregate in TME. Meanwhile, TGF-β, IL-10, IL-35, CCL5, CXCL12 and other immune regulatory factors together form an immunosuppressive microenvironment (5). It is obvious that TME, including a heterogeneous composition of immunosuppressive cells and cytokines, is the critical factor that limits the efficacy of immunotherapy (6). Therefore, exploring the formation of tumor immunosuppressive microenvironment and the underlying molecular mechanisms are of great significance in elucidating the immune tolerance of pancreatic cancer and developing new methods of immunotherapy. In this review, we will focus on the role and the mechanisms of stromal cells in the formation of immunosuppressive TME, as well as the potential clinical application of immunotherapy strategies currently under investigation in targeting tumor microenvironment, aiming to explore new research strategies for pancreatic cancer immunotherapy.

## Formation of immunosuppressive tumor microenvironment in pancreatic cancer

**Figure 1:**
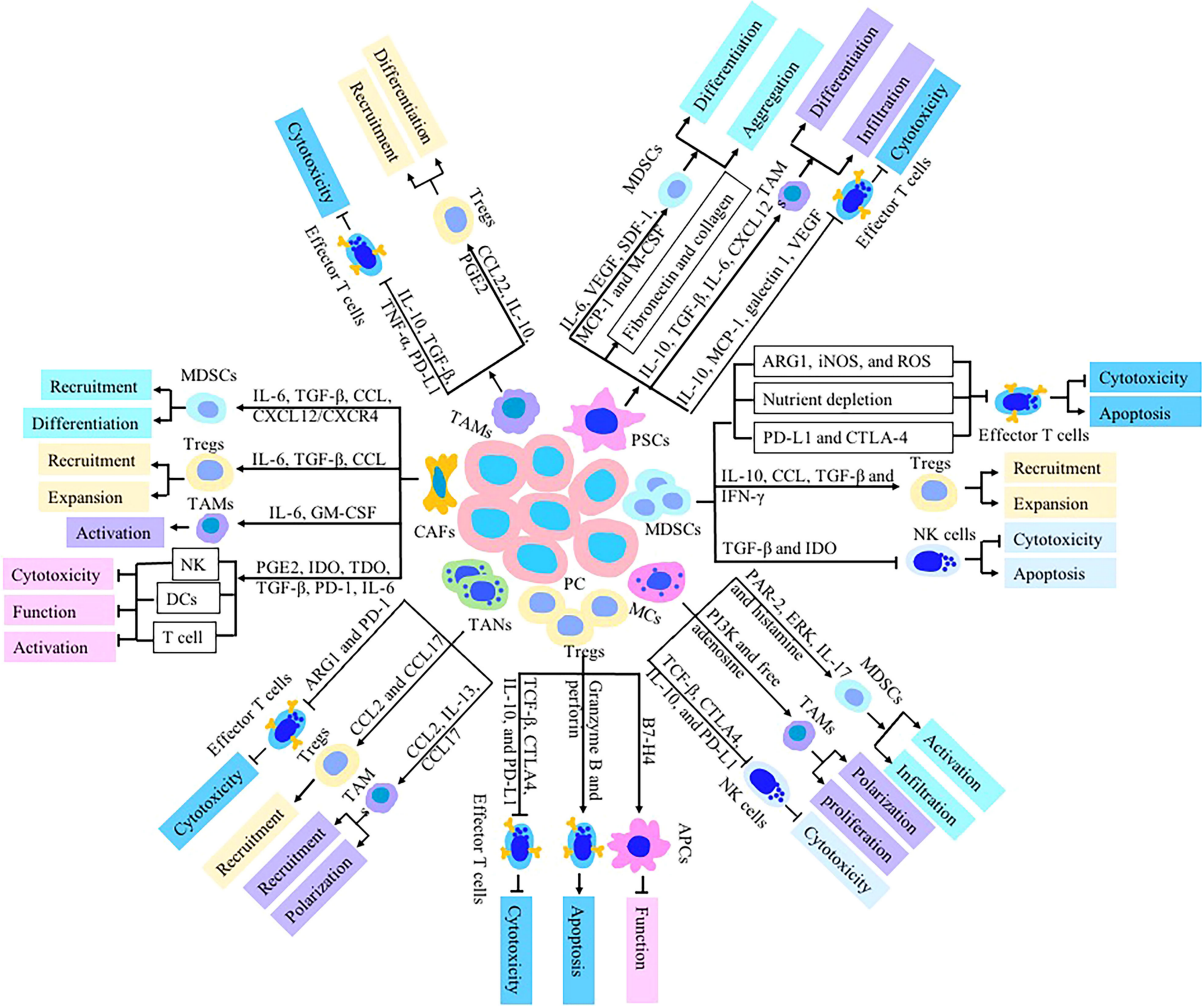
Immunologic effects of stromal cells including MDSCs, CAFs, TAMs, PSCs, Tregs, TANs and MCs on pancreatic TME. These stromal cells dysregulate the function of immune cells-mediated antitumor immunity to establish an immunosuppressive TME *via* secretion of chemokines and cytokines such as TGF-β, IL-10, IL-35, CCL5, CXCL12, GM-CSF, PGE2, IDO, PD-L1 and CTLA-4. The cytotoxic activity and cytokines production of NK cells, DCs and CTLs are inhibited in pancreatic TME.

Various types of immune cells exist in the TME of pancreatic cancer, but these immune cells present abnormality quantity and function, leading to the disorder of the immune function and decrease of anti-tumor ability. The number of CD4^+^, CD8^+^ effector T cells, NK cells and DCs with anti-tumor effects are reduced, and they present nonfunctional status or immature phenotypes. Tregs, CAFs, MDSCs and TAMs with immunosuppressive ability are active and present in large quantities (7). Immunosuppressive cells, cytokines and effector cells’ disability form the immunosuppressive microenvironment of pancreatic cancer, which is an important reason for the insensitivity of pancreatic cancer to immunotherapy. Pancreatic cancer cells secret of various cytokines (such as IL-10, TGF-B and IL-23) and chemokines (such as CXCL1-3, CXCL5, CXCL12, CCI2 and VEGF) to enhance the activation of stromal cells and the accumulation of immunosuppressive cells (8). Activated stromal cells then produce a large number of extracellular matrix and form fibrous basement around pancreatic cancer cells, which hinders the infiltration of effector T cells into tumor (9). Immunosuppressive cells including Tregs, MDSCs and TAMs, up regulate immune checkpoint mediators like programmed death‐1 receptor (PD‐1)–ligand (PD‐L1) and CTL‐associated antigen 4 (CTLA‐4), conferring an inhibitory effect on T cells and NK cells which contribute to form a unique immune suppression tumor microenvironment of pancreatic cancer (10) (**Table 1**). TME become a chief mediator of tumor immune tolerance and immunotherapy resistance (**Figure 1**).

**Table 1:** The effect of stromal components in pancreatic TME on cancer immunosuppression.

Stromal cells	Recruitment & activation	Stromal cell-derived substances	Target cells	Effect on immune response	Ref.
MDSCs	CSF, VEGF, IL, CXCL, TGF-β, TNF-α, IFN-γ, PEG-2	iNOS, arginase, TGF-β, ROS, COX2, IDO, IL-6 and IL-10	NK cells, CD4^+^ and CD8^+^ T, Tregs	Inhibition of lymphocyte activity, recruitment of Tregs and expression of immunosuppressive checkpoint molecules	(11–33)
CAFs	Differentiated from fibroblasts, MSCs, adipocytes and PSCs	IL-6, TGF-β, CCL, CXCL, MCP-1, PGE2, IDO,	Tregs, CD8^+^ T, TAMs, NK cells, tumor cells	Recruitment of Tregs and MDSCs, inhibiting T cells and NK cells activity, and increasing PD-L1 expression	(3, 34–44)
Treg cells	CXCL10, CXCR3-CCL9/10/11, CCR4-CCL17/22 and CCR8-CCL1	TCF-β, CTLA4, IL-10, PD-L1, MADCAM-1, VCAM-1, granzyme B and perforin	NK cells, CD8^+^ T, Th cells, and APCs	Inhibiting the function of immune cells, secreting immunosuppressive cytokines and inducing apoptosis of NK cells,	(45–52)
TAMs	CCL, CXCL, VEGF, TLR4, IL-4, IL-13,	IFN-γ, TNF, iNOS, MHCII, ARG1, IL-10, PGE2, EGF, EGFR, IL-10, TGF-β CD163 and CD204	Tumor cells, CD8^+^ T, Tregs	Inducing Treg differentiation, inhibiting T cells function, producing inhibitory cytokines, increasing the CTLA-4 and PD-1 expression	(53–63)
PSCs	interleukin and TGF-β,	IL-10, CXCL12, MCP-1, VEGF, fibronectin and type I collagen	Immune cells, tumor cells, TAMs, MDSCs,	promoting differentiation and migration of MDSCs and TAMs, leading to imbalance of Th1/Th2 cytokines	(64–68)
TANs	CXCL, TGF-β, IFN-β and GM-CSF	IL-13, CCL17, CCL2, ARG1, elastase and MMP9	Tregs, CAFs, and TAMs	Promoting TAMs polarization, recruitment of Tregs, up-regulating ARG1 and PD-1	(69–72)
DCs	Recruitment is inhibited by PGE2	CD80, CD40, CD70, CD86, MHC-I, MHC-II, IFN-γ, IL-12, IL-15, MGL2 and PD-L2.	T cells, Tregs, Th17.	Impairment of DC activation, maturation and antigen presentation; Inducing the proliferation of Treg cells and inhibiting CD8^+^ T cell- immunity; regulating the balance between Th17 and Treg cells.	
NK cells	HLA-G, CD47, CCL5/CCR5, CCL27/CCR10, CX3CL1/CX3CR1, ECM	GM-CSF, IFN-γ, TNF-α, IL-3, perforin and granzyme	T cells, DCs, macrophages	NK cell toxicity is blocked by MDSCs and Treg cells via TGF-β and inhibitory signals	(73–80)
MCs	FcepsilonRI, TLRs, complement receptors, MMPs and adenosine receptor	IL-13, IL-17 and histamine	MDSCs, Tregs, PSCs, CAFs and NK cells	Promoting the proliferation of CAFs, PSCs and M2-TAMs; Mobilizing MDSCs and Tregs infiltration; Reducing the ligands of NK cell receptor	(81–86)

## The role of stromal cells in immunosuppressive TME formation

### Myeloid derived suppressor cells

MDSCs are heterogeneous cell populations which mainly composed of immature myeloid cells that are found in pathological settings (87). They are precursors of immature monocytes, macrophages, granulocytes and dendritic cells. In patients with pancreatic cancer, the differentiation of monocytes and granulocyte precursors into mature myeloid cells is blocked and they are stimulated by tumor derived factors to differentiate into MDSCs. Studies have shown that MDSCs are mainly distributed in bone marrow, spleen, lymph nodes, peripheral blood and tumor tissues, and gradually accumulate in TME (88). Tumor associated inflammation can stimulate the expansion of MDSCs. Growth factors produced by chronic inflammation (GM-CSF, G-CSF, M-CSF, VEGF and TGF-β), proinflammatory cytokine (IL-1, IL-4, IL-6, TNF-α and IFN-γ) and chemokines (CCL2, CCL4, CXCL1, and CXCL12) can stimulate the expansion of MDSCs (11). These factors induce the amplification of MDSCs *via* JAK/STAT, PI3K/Akt, Notch, NF-κB, NLRP3, Wnt and adenosine receptors A2b signaling pathway, and then MDSCs are recruited to the tumor site by PEG-2, resulting in promoting production of iNOS, arginase, IL-10 and TGF-β (12). Signals that are responsible for converting immature myeloid cells to MDSCs include tumor stroma (proinflammatory cytokines, HMGB1), NF-κB, STAT1, STAT6, COX2 and PGE2 (13). In pancreatic microenvironment, pancreatic stellate cells (PSCs), a subset of pancreatic cancer-associated fibroblasts, promote myeloid cells to differentiate into MDSCs and induce MDSCs to aggregate into TME by producing IL-6, vascular endothelial growth factor (VEGF), macrophage colony-stimulating factor (M-CSF), SDF-1 and MCP-1 in a STAT3-dependent manner (14). Other mediators that are major contributors to induce MDSCs include S100A8 and S100A9 which are regulated by STAT3 and NF-κB. TNF-α also drives the suppressive activity of MDSC by enhancing the binding of S100A8/A9 to the N-glycan motif of receptor for advanced glycation end products (RAGE) (15).

As an important contributor of the immunosuppressive microenvironment in pancreatic cancer, MDSCs induce the inactivation of NK cells, CD4^+^ and CD8^+^ T cells by inhibition of lymphocyte activity, recruitment of Tregs and expression of immunosuppressive checkpoint molecules (16). (1) Direct or indirect inhibition of lymphocyte activity. These mechanisms include elimination of key nutrition needed for T cell proliferation by depleting sequestering L-cysteine and L-arginine, and production of NO and ROS. In the pancreatic TME, MDSCs can compete with T cells to use cysteine which is very important for T cell activation and proliferation, resulting in T cells unable to synthesize *de novo* and hindering the production of anti-tumor T cells (17). In addition, MDSCs also decompose L-arginine and L-tryptophan, further consume essential amino acids and prevent the acquisition of anti-tumor immune cells, resulting in inhibition of anti-tumor immune response. Studies have shown that the expression of arginase 1 (ARG1) and inducible nitric oxide synthase (iNOS) can promote arginine metabolism (18). The high expression of ARG1 and iNOS in MDSCs consumes L-arginine by decomposing L-arginine into L-ornithine and urea. The reduction of L-arginine and the production of nitric oxide in tumor microcirculation can down regulate TCR ζ and MHC II expression, and inhibit T cell activity (19). Release of ROS molecules, which is a critical molecule to maintain the MDSCs in undifferentiated state, is another major mechanism that MDSCs use to suppress T cells by enabling CD8^+^ T cells to lose ability to bind phosphorylated MHC and inducing antigen-specific tolerance of CD8^+^ T cells (20). ROS is formed by superoxide reacting with H_2_O_2_, hypochlorous acid and hydroxyl radical released by MDSCs. H_2_O_2_ inactivate T cells by decreasing T cellular CD3ζ expression and reducing their expression of IFN-γ (21, 22). ROS inhibitors is found to reverse the suppressive effect of MDSCs on T cells to some degree (23). MDSCs also down regulate B cell-mediated immune responses and suppress antibody production by means of arginase, NO and ROS production (24). Under normal physiological conditions, L-tryptophan can produce kynurenine by up regulating the decomposition of indoleamine-2,3 dioxygenase through a STAT3 dependent mechanism. The reduction of L-tryptophan and the production of kynurenine induce the apoptosis of T cells and NK cells, and promote the differentiation of CD4^+^ T cells into Tregs (25). MDSCs just stand at the top of this regulatory chain and consume L-tryptophan, which promotes the accumulation of canine urinary ammonia, and finally leads to the promotion of immunosuppression of tumor microenvironment. (2) Stimulatory effect on Tregs, which is one important mechanism of MDSC-mediated T cell inhibition. Immunosuppressive cytokines (IL-10), chemokine ligand (CCL4 and CCL5) and TGF-β produced by MDSCs can induce Tregs into the TME. MDSCs also promote the expansion of induced Treg cells through TGF-β, CD40/CD40L, IL-10 and IFN-γ (26). CD40/CD40L signal pathway plays a role in the homeostasis of Tregs and the homeostasis is disrupted under pathologic conditions. It has been proven that expression of CD40 on MDSCs is critically important for MDSCs-induced Treg proliferation. The involvement of CD40/CD40L in the interplay between MDSCs and Treg in the presence of TGF-β and IL-10 promotes activation and expansion of Treg. Interference with CD40/CD40L interactions reverse the suppression of T-cell responses and inhibits Tregs activity (27, 28). (3) Expression of immunosuppressive checkpoint molecules. PD-1/PD-L1 and CTLA-4 are the best-known co-inhibitory pathways. MDSCs express a high level of PD-L1, which binds to PD-1 expressed on T cells, induces conformational changes, and then makes T cells inactive and dysfunctional (29). Under hypoxia conditions in TME, hypoxia inducible factor 1-α (HIF1-α) selectively up-regulate the expression of PD-L1 on the surface of MDSCs by binding to the hypoxia-response element (HRE) in the PD-L1 proximal promoter (30). Blockade of PD-L1 expression abrogate T cell suppression regulated by MDSCs *via* modulating production of IL-6 and IL-10 (30). In addition, the ARG1 secreted from MDSCs promote the binding of inhibitory checkpoint ligands with receptors expressed on T cells, accelerating the depletion of T cells, and this effect is abrogated by arginase inhibitor (31). (3) Immunosuppressive cytokines secreted by MDSCs are important factors to inhibit antitumor immune response in pancreatic cancer. The factors involved in MDSC-mediated immune suppression include TGF-β, COX2, indoleamine 2,3-dioxygenase (IDO), IL-6 and IL-10 *via* NF-κB and STAT3 signaling pathways (32). TGF-β derived from MDSCs plays a key role in inhibiting antitumor immune response. As immunosuppressive cytokines, TGF-β inhibit the activity of cytotoxic T cells and NK cells by reducing IFN-γ. The anti-TGF-β monoclonal antibody restores T-cell anti-tumor response and IFN-γ production to a certain degree (33). Intimate interactions exist between MDSCs and other components such as TAMs, NK cells, CAFs, and T cells in the TME. Due to suppressive activities against effector lymphocytes in TME, MDSCs act as a major barrier to pancreatic cancer immunotherapy. Various therapeutic approaches targeting MDSCs including immunostimulatory adjuvants, therapeutic blockade of the mobilization and survival of MDSCs, and anti-inflammatory agents are being tested in preclinical and clinical studies.

### Tumor associated fibroblasts

CAFs in TME are heterogeneous cell populations with different origins. Bone marrow-derived fibroblasts and locally infiltrated fibroblasts are considered to be the major sources of CAFs. Mesenchymal stem cells, adipocytes and PSCs can also be transformed into CAFs (34). Various cellular precursors of CAFs result in fibroblast heterogeneity. Four different CAFs populations (referred as CAF-S1 to CAF-S4) have been identified recently by combining the analysis of six CAFs markers including smooth-muscle α-actin (SMA), fibroblast activated protein (FAP), fibroblast specific protein-1 (FSP1, also known as S100A4), integrin β1 (CD29), platelet derived growth factor receptor (PDGFR) and podoplanin (PDPN) (35). As an important part of pancreatic TME, CAFs actively affect nearly all aspects of cancer biology including tumor growth, immunosuppression, invasion and angiogenesis. CAFs promote tumor immune escape through secretion of cytokines, chemokines, metabolites and enzymes, and recruitment of inhibitory immune cells. Cytokine (e.g., IL-6 and TGF-β) and chemokine (e.g., CCL2, CCL5, CXCL1 and CXCL12) released from CAFs attract suppressive immune subsets including MDSCs and Tregs into TME (36). Inducing monocytes to differentiate into M2 macrophages by CAFs is mainly mediated *via* CXCL12/stromal cell-derived factor 1 (SDF1), M-CSF/CSF-1, IL-6, IL-8 and CCL2/monocyte chemotactic protein-1 (MCP-1). CAFs enhance pro-tumoral M2-TAMs activation *via* increased secretion of IL-6 and GM-CSF in response to cancer cell stimulation (37). Then CAFs coordinate and synergize with M2-TAMs to inhibit the activity of T cells. The proportion of TAMs and polymorphonuclear MDSCs (PMN-MDSCs) in pancreatic TME is decreased significantly through inhibiting colony stimulating factor 1 receptor (CSF-1R) and CXC chemokine receptor 2 (CXCR2). CAFs recruit monocytes into TME through the CXCL12/CXCR4 and CCL2 pathway, and induce their differentiation into MDSCs through IL-6 -STAT3 signal axis (38, 39). CAFs attract Tregs and decrease CD8^+^ T cells infiltration into TME by secreting TGF-β and IL-6, thus interfering with tumor-directed immune response (40). In pancreatic cancer, CAFs down regulate NKp30, NKp44, NKG2D, perforin and granzyme B expressed on NK cells by secreting prostaglandin E2 (PGE2), IDO and TGF-β, thereby decreasing NK cell cytotoxic activity (41, 42). Another mechanism by which CAFs trigger an immunosuppressive response in TME is by inhibiting the function of DCs. TGF-β secreted by CAFs induces DCs to down regulate the major histocompatibility complex (MHC) class II molecules and costimulatory molecules such as CD40, CD80 and CD86, thus decreasing their ability to activate cytotoxic T cell responses and their antigen presenting function (35). Activated CAFs express tryptophan-2,3-dioxygenase (TDO), promote the degradation of tryptophan and inhibit the function of DCs. CAFs kill directly CD8^+^ T cells or reduce CD8^+^ T population through secreting PD-1, Fas ligand (FasL) and IL-6, and inhibiting the expression of B cell lymphoma 2 (Bcl-2) (3, 40). CAFs have immune regulatory effects on tumor cells, such as up regulating the expression of immune markers on tumor cells. CAFs increase the expression of PD-L1 in pancreatic cancer by secreting CXCL5 and CXCL12 *via* phosphatidylinositol-3-kinase/protein kinase B signaling pathway, thus promoting the formation of immunosuppressive microenvironment (43, 44). However, Costa et al. revealed the fibroblast heterogeneity and showed that CAF-S1 promotes an immunosuppressive microenvironment by recruiting CD4^+^CD25^+^ T cells, while another subtype, CAF-S4, do not exhibit the properties. CAF-S1 is associated with accumulation of FOXP3^+^ T Lymphocytes and enhances regulatory T cell differentiation through B7H3, CD73, and DPP4 (89). Both CAF-S1 and CAF-S4 promote metastases through complementary mechanisms (90). FAP^High^ CAFs attract CD4^+^ CD25^+^ T lymphocytes and retain them through high expression of OX40 L/TNFSF4, PD-L2/PDCD1LG2 and JAM2 (91). FAP^High^ CAFs secrete high levels of TGF-β ligands to promote cell death of effector CD8^+^ T lymphocytes by inhibiting the expression of the pro-survival protein BCL2 (92). α-SMA^+^ FAP^+^ CAFs decrease the proliferation of CD8^+^ T cells and induce the recruitment of CD4^+^ CD25^+^ T cells *via* secreting TGF-β and IL-6 (93). Both ecm-myCAF and TGF-ß-my CAF exhibit an immunosuppressive function and contribute to resistance to immunotherapy *via* recruiting FOXP3^high^ Tregs and increasing PD-1 and CTLA-4 protein expression (94). α-SMA^+^ CAFs are positively correlated with PD-L1 expression by abolishing T cell anti-tumor immune response (95). The amounting evidence confirms that specific CAF subpopulation functions is complex with the different specific markers, which is one of the most challenges in the study of CAFs. The immunosuppressive effect of CAFs on tumor cells reflects that CAFs can become a therapeutic target in pancreatic cancer immunotherapy. Many drugs targeting key regulator of CAFs are undergoing clinical and/or preclinical evaluation. At present, anti CAFs treatment mainly focuses on fibroblast activation protein (FAP). As a specific marker of CAFs, FAP is a transmembrane serine protease, which is highly expressed on CAFs in most epithelial tumors. In the pancreatic cancer, the deletion of FAP gene can significantly reduce the infiltration of FAP^+^ CAFs and induce tumor hypoxic necrosis (96). Elimination of FAP^+^ CAFs by DNA vaccination and chimeric antigen receptor (CAR)-T cells has become an important immunotherapy strategy (97). A study showed that oral FAP-DNA vaccine could induce both CD8^+^ and CD4^+^ immune responses, and antitumor immunity was enhanced by combing FAP DNA vaccine with other tumor antigen-specific DNA vaccines (98). However, it should not be ignored that bone marrow mesenchymal stem cells and skeletal muscle expressing FAP may also be recognized and impaired by FAP specific CAR-T cells, leading to fatal bone marrow toxicity and cachexia. In addition to directly eliminating and inhibiting CAFs, it is also effective to restore CAFs to normal state. Angiotensin receptor blockers can reprogram CAFs into to a quiescent state, alleviate immunosuppression and improve T lymphocyte activity (99). but its clinical application is limited because of systemic adverse reactions. Resetting activated CAFs into the static state through vitamin A and vitamin D has attracted extensive attention. Administration of all-trans-retinol can inhibit activated CAFs that promote tumor signal pathway, significantly increase CD8^+^ T cell infiltration, and improve the efficacy of cancer chemotherapy (100–102). A randomized, double-blind, placebo- controlled trial performed by Morita et al. reported that Vitamin D supplementation decreased serum levels of PD-L1 in digestive tract cancer and significantly reduced the risk of relapse/death in the highest level of PD-L1 (103). Due to the toxic effects of directly targeting CAFs, researchers begin to focus on the effector molecules that inhibit the secretion of CAFs. Targeting CAFs related signaling pathways with immunotherapy is a possible approach for improving the anti-tumor efficiency. Blocking cytokines secreted by CAFs (such as IL-6, IL-8, IL-10 and TGF-β) combined with PD-1/PD-L1 can improve the anti-tumor immune response through increasing T cell infiltration and alleviating the role of PD-1 in inhibiting T cell activity (3). Thus, targeting CAFs related signaling pathways may be a new strategy for the treatment of pancreatic cancer.

### Regulatory T cells

Treg cells are a kind of T cell subsets that regulate a variety of immune cell functions. They play a critical role in suppressing anti-tumor immune response. Treg cells maintain the balance of the immune system and avoiding autoimmune diseases, allergic reactions and transplantation rejection under physiological conditions (104). In patients with pancreatic cancer, Treg cells are an important component involved in the inhibition of tumor immune response by disordering the functions of CD4^+^ T and CD8^+^ T cells, macrophages, NK cells and DC cells (105, 106). The number of Treg cells in pancreatic TME increases significantly, and is correlated with tumor metastasis and poor prognosis (45). The infiltrated Treg cells were directly proportional to the infiltrated CD4^+^ T cells and inversely proportional to the infiltrated CD8^+^ T cells. Treg cells reduce the infiltration of antitumor effector lymphocytes in peritumoral tissues by secreting IL-35. Treg cells are chemoattracted to TME by chemokines including CXCR3-CCL9/10/11, CCR4-CCL17/22 and CCR8-CCL1. Tumor cells secrete cytokines such as CCL5, CCL17 and CCL22 which Treg are chemotactic to (46). In addition, studies have shown that pancreatic cancer cells induce PSCs to express and secrete CXCL10 which acts as a chemoattractant for Treg cells. CXCL10 mediate the recruitment of CXCR3^+^Treg cells to tumors *via* its cognate receptor CXCR3 (47). Treg cells secrete inhibitory cytokines such as TCF-β, CTLA4, IL-10, and PD-L1 to mediate the immune tolerance of the effective cell to pancreatic cancer associated antigen, thereby avoiding the tumor cells from immune surveillance. Pancreatic cancer with higher Treg and lower Th17 cells alter cytokine IL-10, IL-17, IL-23, INF-γ and TGF-β expression by regulating transcription factors such as CTLA-4, RORγt, ROR-α, and FoxP3 (48). Other studies have found that tumor derived endothelial cells highly express mucosal epithelial cell adhesion molecule-1 (MADCAM-1), vascular cell adhesion molecule-1 (VCAM-1), and the ligands of these adhesion molecules are specifically expressed on Treg cells. Specifically blocking the adhesion molecules reduce the adhesion of Treg cells to tumor derived vascular endothelial cells and reduce the infiltration of Treg cells into TME (49). Cytokines secreted by cancer cells and other stromal cells in pancreatic tumor microenvironment, such as TGF-β1 induce the differentiation of CD4^+^ CD25^-^ T cells into CD4^+^ CD25^+^ Foxp3^+^ Treg cells. Application of TGF-β neutralizing antibodies can block this differentiation process (50). Treg cells inhibit the function of immune cells such as CD8 cytotoxic T lymphocytes, CD4 helper T cells and NK cells. However, the mechanism is still controversial. Experimental data supports that it mediates the inhibitory effect on immune response mainly by secreting immunosuppressive cytokines such as TGF- β and IL-10. Treg cells induce antigen presenting cells (APCs) to express B7-H4 by triggering high levels of IL-10 production, and then these APCs expressing B7-H4 inhibit the proliferation of T cells by interacting with the corresponding receptors (51). Activated Treg cells directly induce apoptosis of NK cells and CD8^+^ T cells by secreting granzyme B and perforin. In addition, Treg cells combine with APCs to induce the expression of IDO, so as to inhibit the activation of T cells (52). In the pancreatic TME, tumor cells are conferred with the biological functions of the immune cells. They cooperate with infiltrating Treg cells and macrophages to play an immunosuppressive function and promote tumor progression.

### Tumor associated macrophages

TAMs are important inflammatory cells in the pancreatic TME. Tumor cells secrete chemokines (CCL2, CCL7, and CXCL12) and cytokines (VEGF) to recruit peripheral blood monocytes into tumor tissues and induce them to differentiate into TAMs. Macrophages can be divided into M1 type and M2 type. The former, which is induced by Toll-like receptor 4 (TLR4) and IFN-γ, secrete tumor necrosis factor (TNF), iNOS and MHC class II molecules, and has the ability to coordinate the anti-tumor immune response of Th cells; M2-macrophages are induced by IL-4 and IL-13 which secreted by CD4^+^ T cells. M2-macrohphages with high level of ARG1, IL-10, CD163 and CD204 expression have anti-inflammatory responses and pro-repair, pro-tumoral and anti-parasitic ability (53). Macrophages in a state of constant transition between the two forms of M1 and M2 phenotype adopt context-dependent phenotypes when stimulated. They are differentiated and activated mainly by IRF/STAT (interferon-regulatory factor/signal transducer and activator of transcription) signaling pathway (54). Tumor associated macrophages (TAMs) which are broadly considered M2-like secrete a large number of cytokines including PGE2, epithelial growth factor (EGF), epithelial growth ligands of the factor receptor (EGFR), IL-10 and TGF-β, which stimulate tumor cell proliferation and survival (55). C5aR1 which is generally considered to be proinflammatory can induce macrophages to differentiate into M2 type *via* activation of NF-κB pathway (56). C1q characterizes macrophages with a tolerant or immunosuppressive phenotype, which lead to the up regulation of PD-L1 and PD-L2 and the inhibition of T cell proliferation. C1q also has a direct effect on T cells by regulating the mitochondrial metabolism of CD8^+^ T cells (57). TSC-mTOR pathway which acts as central interchange for cell proliferation switches macrophages into M2-like TAMs to promote immune-suppressive TME (58).

TAMs take precedence in the pancreatic TME leading to immune evasion through production of inhibitory cytokines (IL-10 and TGF-β), metabolic activities consisting of depletion of L-arginine, ROS production and immune checkpoint engagement. TAMs secrete TNF-α and IL-10 to promote the expression of PD-L1, thereby inhibiting the function of antitumor T cells (59). TGF-β from TAMs promote TAMs to secrete CCL22 and then recruit Treg into TME; Moreover, TGF-β also increase the expression of CTLA-4 and PD-1 on the surface of CD8^+^ T cells, and reduce the production of granzyme and IFN. TAMs secrete chemokines such as CCL to recruit Tregs into TME, and produce IL-10 and PGE2 to induce Treg differentiation in TME (60). Moreover, Zhou et al. reported that exosomes played a critical role in the interaction between TAMs and T cells. Exosomes including miR-29a-3p and miR-21-5p derived from TAMs directly suppressed STAT3 and induced an imbalance Treg/Th17 cells, generating an immune suppressive microenvironment (61). TAMs upregulated the expression of B7-H4 on the surface of cancer cells through EGFR/mitogen activated protein kinase (MAPK) pathway, and attenuated the cytolytic effect of T cells (62). TAMs in pancreatic TME can serve as a potential target for immunotherapy. IFN-γ possesses immunoregulatory and anti-tumor properties. IFN-γ directly converts TAMs to the M1 subtype. IFN-γ enhances the efficacy of PD1 blockade therapy by inhibiting CXCR2-expressing M2 TAMs tumor trafficking and infiltration (63). A deeper understanding of roles of TAMs in immunotherapy will help explore the targeting TAMs as an adjuvant therapy in tumor immunotherapies.

### Pancreatic stellate cells

As one of the important sources of CAFs in pancreatic cancer, PSCs is a fibroblast like cell containing vitamin A lipid droplets in the cytoplasm which secrete extracellular matrix (ECM) components and various cytokines (64). Quiescent PSCs can be activated by cytokines such as interleukin and TGF-β, and the activated PSCs secrete soluble cytokines, including IL-10, MCP-1 and VEGF which interact with immune cells in TME, resulting in the increasing the number of immunosuppressive cells and decreasing the infiltration of anti-tumor immune cells in TME. Moreover, activated PSCs produce large amounts of ECM components such as fibronectin and type I collagen, which form a barrier to immune cell infiltration and facilitate pancreatic cancer cells immune escape (65). Inhibiting the secretion of matrix by PSCs can increase the transport efficiency of chemotherapeutic drugs in TME and improve the response of pancreatic cancer to immunotherapy. Dynamic and bidirectional crosstalk exists between PSCs and other stromal cells in the pancreatic TME. PSCs and macrophages interact with cytokines reciprocally. PSCs induces macrophages to differentiate into M2 type with elevated expression of IL-10, TGF-β, PDGF-β, CD206, and CD301 and decreased expression of M1 macrophage marker iNOS. M2 macrophages increase the expression of TGF-β and PDGF which promote the proliferation and activation of PSCs (66). PSCs promote the differentiation and migration of MDSCs into TME by activating IL-6 and CXCL12. In addition, IL-6 and CXCL12 also promote the infiltration of Treg into TME. PSCs promote the apoptosis of CD4^+^ and CD8^+^T cells in pancreatic cancer by secreting galectin 1 (67). PSCs reduce the secretion of Th1 cytokines such as IL -2 and IFN-γ, and enhance the secretion of Th2 cytokines including IL-4 and IL-5, resulting an imbalance of Th1/Th2 cytokines. Further elucidation of the role of PSCs in immunosuppression could lead to exploring novel therapeutic targets in pancreatic cancer (68).

### Tumor associated neutrophils

A large amount of TANs infiltrate in the pancreatic cancer. The crosstalk between tumor cells and TANs drive tumor growth and progression. TANs have tumor promoting effects and is associated with poor prognosis of pancreatic cancer *via* immunosuppression and regulation of other immune cell populations. TANs are classified as N1 (antitumor) or N2 (protumor) according to their activation, cytokine status and effects on tumor cell growing (107). In pancreatic TME, stromal cells and cytokines can regulate the recruitment, activation and differentiation of neutrophils. CAFs participate in the recruitment of neutrophils to TME, especially by secreting CXC1, CXCL2, CXCL5, CXCL6, CXCL2 and chemokine ligand 2. Tumor-derived cytokines, such as TGF-β and GM-CSF extend survival of TANs in TME. TGF-β in TME secreted by CAFs induces neutrophils to differentiate into N2 TANs, which in turn recruit Treg cells through CCL17 secretion (69, 70). When IFN-β or TGF-β in TME is inhibited, TANs show the N1 phenotype of tumor inhibition. When TGF-β is increased at high levels, TANs showed the N2 phenotype of immunosuppression and cancer promotion, and their gene expression patterns also changed, such as up regulation of CCL17, ARG1 and MMP9. N2 neutrophils leads to somatic DNA instability and promotes tumorigenesis by producing ROS, NO and PEG2. Zhou et al. reported that TANs recruited macrophages and Treg cells to infiltrate the tumor site by secreting CCL2 and CCL17, and contributed to sorafenib resistance. The number of infiltrated TANs could be used as a biomarker for predicting responsiveness to sorafenib (71). TANs also accumulate in tumor tissues and secrete elastase, which promotes EMT and secretion of MMP-9 in pancreatic cancer, and promotes tumor invasion and angiogenesis. Moreover, TANs inhibit antitumor immunity by up regulating ARG1 and PD-1. There exists mutual influence between TANs and TAMs and they coordinate effects within the TME. N2 TANs promotes TAMs polarization *via* secretion of IL-13 (72). TANs and TAMs secrete CCL17 to induce the recruitment of Treg cells to the TME. The interaction between TANs and TAMs requires targeting common TAM and TAN signal pathway as a means to enhance the efficacy of current therapies.

### Dendritic cells

Dendritic cells (DCs), as one of the most powerful APCs in the immune system, are central regulators of the adaptive immune response and essential for T cell-mediated cancer immunity (108). DCs express co-stimulatory molecules (CD80, CD40, CD70 and CD86) and antigen-presenting molecules such as MHC-I and MHC-II, and DCs deliver the co-stimulatory signals to activate T cells. DCs also enhance T cell expansion and polarization to secret of IFN-γ *via* cytokine signals including IL-12 and IL-15 (109). Therefore, DCs play a critical role in anti-tumor and immunoregulatory activities. The study of DCs infiltrated in pancreatic TME is of great significance to understand the mechanism of tumor immune evasion. CD103^+^ DCs in tumors are necessary to recruit effector T cells into the TME and priming tumor-specific CD8^+^ T cells. It is reported that the recruitment of effector T cell subsets into TME depended on the expression of CXCL9 and CXCL10 which mainly released from CD103^+^ DCs in tumors (110). Abound infiltration of CD103^+^ DCs in tumor improve responses to therapeutic PD-L1 and BRAF blockade (111). However, A network of immunosuppressive factors in TME inhibit DCs infiltration and their anti-tumor activity. Tumor decrease DCs infiltration by reducing CCL4 expression and producing PGE2. DCs infiltrated in tumor enhance expression of T cell immunoglobulin mucin receptor 3 (TIM3) which sequesters high mobility group protein B1 (HMGB1), resulting in impairment of DC activation and antigen presentation (112). Metabolites in the TME such as lactic acid which is a major metabolic product of tumor cells dampen DCs differentiation and activation. DCs interact with Treg cells to induce immune tolerance. Treg cells prevent the expression of CD80 and CD86 to inhibit the maturation of DCs (113). In addition, Treg cells regulate the cytokines produced by DCs. For example, Treg cells decrease IL-6 and increase IL-10 expression in DCs. In metastatic pancreatic cancer, DCs induce the proliferation of Treg cells and inhibit CD8^+^ T cell-mediated tumor immunity *via* MGL2 and PD-L2, establishing an immunosuppressive microenvironment favor for metastasis formation (114). DCs also regulate the balance between Th17 and Treg cells. Some cytokines in TME, such as macrophage colony stimulating factor, IL-4, IL-6, IL-10, TGF-β and VEGF can alter the differentiation of DCs and inhibit the activation and maturation of DCs, resulting in a lack of mature and functional DCs in tumor patients. These immature DCs do not express or down regulate costimulatory molecules, MHCII molecules and antigen presentation related transporters, but express indoleamine 2,3-dioxygenase, which degrade tryptophan and inhibit T cell immunity (115). Surgical resection, chemoradiotherapy or immunochemotherapy can increase the number of DCs in the circulation and restore its function to some extent. Increased DCs not only prolong the survival, but also significantly reduce the risk of postoperative infection complications in patients undergoing pancreatectomy. These findings suggest that immunotherapy to increase the number of DCs and restore their function is beneficial to improve the prognosis of patients (116).

DC vaccine is the focus of tumor immunotherapy. The treatment strategy based on DCs is to use patients’ own DCs to produce therapeutic vaccine. First, DCs are collected from the patient, matured *in vitro*, loaded with tumor antigen and injected back into the patient. After injection, DCs present tumor antigen to T cells, leading to T cell activation and initiating T cell response. DC vaccine has been proved to be safe for the treatment of pancreatic cancer (117). It is confirmed that the combination of gemcitabine and DC vaccine can promote the recruitment of CD8^+^ T cells and enhance CTLs mediated tumor cell death in a mouse model of pancreatic cancer (118). Recent multicenter clinical studies have also confirmed that DC vaccine and chemotherapy drugs induced to produce tumor antigen-specific CTLs, having a synergistic effect on pancreatic cancer patients (119). In order to restore the function of DCs in TME, many attempts have been made. It is reported that impaired function of DCs as well as the immunosuppressive effect of Treg can be reversed by activating TLR-7/8 and TLR-9 signal pathways (120). Because the suppressor of cytokine signaling 1 (SOCS1) is a negative regulator that inhibit the maturation of DCs, some studies have designed a siRNA to interfere with SOCS1, aiming to promote the antigen presentation of DCs, activate TLRs signal pathway and promote the maturation of DCs (121).

### Natural killer cells

Natural killer (NK) cells are an important part of tumor immunosurveillance with ability of directly killing cancer cells or indirectly promoting the immune responses. NK cells secrete a large number of cytokines including GM-CSF, IFN-γ, TNF-α and IL-3 which affect the activity of other immune cells such dendritic cells, neutrophils and macrophages, thus influencing the subsequent T and B cell responses (73). Chemo-attractants/receptors (including CCL5/CCR5, CCL27/CCR10 and CX3CL1/CX3CR1) and immunomodulation of chemokine axes (including HLA-G and CD47) are involved in controlling NK cell recruitment to the tumor. Extracellular matrix (ECM) barriers also play a role in regulating NK cells infiltration into TME (74). The activation and functional status of NK cells are depended on interacting signals between activating co- stimulatory and inhibitory signals. Activating signals mainly comprise integrins, killing-receptors (CD16, NKp30, NKp40 and NKp44), and other receptors such as NKp80, SLAMs, CD18, CD2 and TLR3/9. Inhibitory signals include Ly49s, NKG2A and LLT1 (Summarized in (75)). NK cells kill or ignore target cells based on the balance between ligands expressed on tumor cells that mediated inhibitory and activated signal. NK cells induce apoptosis of cells through producing a huge amount of IFN-γ, perforin and granzyme family molecules after re-stimulation. The sensitivity of target cells to NK cells mediated cell lysis mainly depends on the expression of MHC I. NK cells selectively lyse cells with low expression of MHC type I molecules or cells that lose the expression of MHC I. Virus infected cells or tumor cells down regulate the expression of MHC molecules, and become possible targets for NK cells (76). The cytotoxic ability of NK cells can be enhanced by IL-2, IL-12, IL-15 and IFN-α/β) while their cytotoxic functions are inhibited by immunosuppressive factors such as TGF-β, IL-10, PGE2, and IDO1/2 (75). Close interaction and a positive feedback loop exist between macrophages and NK cells in the TME. NK cells activate macrophages and promote M1-like macrophage polarization to secrete a large number of cytokines such as IL-12/18 by expressing IFN-γ, TNF-α and GM-CSF. Meanwhile, IL-12/-18 secreted by activated macrophages increase of NK cell cytotoxicity and IFN-γ production, and enhance the expression of CD80, CD86, HLA-DR and HLA-DQ (77). The interaction between NK cells and DCs can lead to the activation of NK cells, cytokine production, cytotoxicity and the maturation of DCs (78). NK cell toxicity, IFN-γ production and NKG2D expression are blocked by MDSCs and Treg cells in a TGF-β-dependent mechanism in pancreatic TME. TGF-β is also able to decrease NKp30- and NKG2D- expression on NK cells, thus interfering possibly the crosstalk of NK cells and DCs (79, 80). In the TME of pancreatic cancer, the receptor NKG2D, NKp30 and NKp46 related to NK cell activity are significantly downregulated. Therefore, the number of NK cells in pancreatic cancer tissue is decreased significantly. However, due to heterogeneity of NK cells and complexity of tumor intrinsic signal pathways, the specific role of NK cells in cancer immune response is depends on distinct cancer types and need further elucidation.

### Mast cells

Mast cells derived from CD34^+^/CD117^+^ pluripotent hematopoietic stem cells release chemokines and cytokines after activation. MCs activation is triggered by a variety of receptors such as high-affinity IgE receptor (FcepsilonRI), TLRs, complement receptors and adenosine receptor (81). MCs are recruited to the TME by pancreatic cancer cells, and the infiltrated MCs promoted pancreatic cancer progression in a matrix metalloproteinase (MMPs) -dependent manner (82). Moreover, there is complex interaction between mast cells and stellate cells in pancreatic cancer. Stellate cells activate mast cells to secrete IL-13 and tryptase which promote the proliferation of CAFs and PSCs, establishing a feed-forward loop between MCs and PSCs (83). MCs activated CAFs and TGF-β signal pathway to increase the resistance to Gemcitabine/Nabpaclitaxel and promote tumor invasion in pancreatic cancer *via* PAR-2, ERK1/2 and Akt activation (84). Inhibiting MCs with AMD3100 suppresses tumor growth. Activated MCs inhibit the anti-tumor immune response. MCs promote M2-TAMs polarization and proliferation by PI3K and free adenosine (85). MCs mobilize MDSCs infiltration into tumors and induce MDSCs to secrete IL-17 or histamine, then recruiting Tregs into pancreatic TME and enhancing their suppressive function (86). Histamine reduce the ligands of NK cell receptor expressed on tumor cells and interfere with the recognition and cytotoxicity of tumor cells mediated by NK cells. New possibilities for therapeutic intervention based on MCs need further exploration of the crosstalk between MCs and stromal cell in pancreatic TME.

## Immunotherapy strategy for pancreatic cancer based on tumor microenvironment

Stromal cells and cytokines in pancreatic TME promote tumor growth and metastasis by inhibiting the anti-tumor immunity and enhancing the activity of immunosuppressive cells. It is a problem to be solved in tumor immunotherapy that how to reprogram this environment conducive to tumor growth so that the anti-tumor immunity can be restored. New immunotherapy for cancer is expected to be a breakthrough in the treatment of pancreatic cancer. Immunotherapy is rated as the first of the top ten scientific and technological breakthroughs in 2013 because of its remarkable effect on tumor which strongly proves the future potential of tumor immunotherapy (122) Investigations into an increasing variety of immunotherapies and combing with other traditional chemoradiotherapy are urgent need for more effective therapeutic strategies. Combination therapy approaches, such as combining immune checkpoint inhibitors with chemotherapy or radiation therapy, may be necessary to achieve optimal outcomes in this disease. Immunotherapy strategy for pancreatic cancer based on tumor microenvironment is summarized in **Table 2**. From the perspective of clinical application, it is important to find a biomarkers to identify patients who are likely to respond to immunotherapy. Currently, there is no established biomarker for predicting response to immunotherapy in pancreatic cancer, and this has limited the success of immunotherapy. More research is needed to identify biomarkers that can predict response to immunotherapy, such as tumor mutational burden, microsatellite instability, and immune cell infiltration, which could help guide patient selection and treatment decisions.

**Table 2:** Immunotherapy strategy for pancreatic cancer based on tumor microenvironment.

Intervention	Strategy	Cancer stage	Trials/Identifier	Outcomes & response	Ref.
ICI					
Ipilimumab	CTLA-4 antibody	Advanced PC	Ib	Gemcitabine and ipilimumab is a safe regimen with tolerable adverse events	(123)
Pembrolizumab	PD-1 antibody	Metastatic PC	I/II NCT02331251	Combining pembrolizumab with gemcitabine and nab-paclitaxel chemotherapy improve the prognosis.	(124)
Tremelimumab	CTLA-4 antibody	Metastatic PC	I/ NCT00556023	The median OS was 7.4 months (95% CI 5.8-9.4 months) by combing tremelimumab plus gemcitabine with tolerable toxicities	(125)
Tremelimumab	CTLA-4 antibody	Metastatic PC	II/NCT02527434	The efficacy of tremelimumab as monotherapy was unsatisfactory with a poor prognosis	(126)
^Targeting TME^					
Galunisertib	TβRI kinase inhibitor	Unresectable PC	1b/II	Galunisertib combining with gemcitabine improve the OS and PFS	(127)
Galunisertib	TβRI kinase inhibitor	Metastatic PC	Ib /NCT02734160	Combining galunisertib with durvalumab achieved a DCR of 25.0% and a confirmed ORR of 3.1%	(128)
PECPH20	Intratumoural pressure	Animals models	Preclinical studies	PECPH20 combined with chemotherapeutic drugs decrease tumor volume and improve OS	(129)
PECPH20	Intratumoural pressure	Metastatic PC	IB/II	This combination of PECPH20 with FOLFIRINOX caused increased toxicity with shortened OS.	(130)
PECPH20	Intratumoural pressure	IV PC	Ib	OS and PFS were not improved in patients treated with PECPH20 combined with paclitaxel and gemcitabine	(131)
Bruton tyrosine kinase inhibitor	MDSCs	Metastatic or locally advanced PC	II	The combination of acalabrutinib and pembrolizumab was well tolerated with disappointing ORR and DCR	(132)
INF-2α	IFN-γ and IL-10	Resected PC	NP	INF-2α promoted the DCs and NK cells activation	(133)
IL-6	Targeting myeloid progenitor cells and B and T lymphocytes	Advanced PC and colon cancer	I	Induction of CRP and IgE; inhibition of NK and lymphokine-activated killer cell activity	(134)
BL-8040	CXCR4 antagonist	metastatic PC	NCT02826486	Combined BL-8040 and pembrolizumab improved the effect of chemotherapy	(135)
Cancer vaccination					
DC vaccination	MUC1 peptide-loaded DCs	Metastatic PC positive for MUC1	I	The MUC1-peptide-pulsed DCs was non-toxic and capable of inducing immunological response	(136)
DC vaccination	MUC1 peptide-loaded DCs	Unresectable or recurrent PC		MUC1-DC was feasible and effective with tolerable toxicity	(137)
DC vaccination	MUC1 peptide-loaded DCs	Resected PC	I/II	The median survival is 26 months (range 13-69 months) for all patients with	(138)
DC vaccination	DC/WT1-I/II	IV PC	I	The DC/WT1-I/II combined with chemotherapy effectively activated WT1-specific immune responses and promoted disease stability	(139)
DC vaccination	Poly-ICLC to peptide-pulsed DCs	Advanced PC	NCT01410968	DCs loaded with poly-ICLC is safe and induces a measurable tumor specific T cell population	(140)
NK cells	Irreversible electroporation (IRE)	Metastatic PC	NCT02718859	Combining NK cells with IRE had a synergistic effect with satisfactory short-term outcome	(141)
NK cells	(IRE)	III/IV PC	NP	Combining NK cells with IRE significantly extended PFS and OS	(142)
Lenalidomide	NK cells	Advanced PC	NCT01547260	Lenalidomide augmented a significant increase in the numbers of CD4^+^ and CD8^+^ T cells	(143)
KWAR23	SIRP α monoclonal antibody	Solid tumors	NP	Enhancing myeloid cell-dependent tumor killing ability, activates neutrophils and macrophages, and inhibits tumor growth	(144)
Extracellular vesicles (EVs)					
Exosomes from fibroblast-like mesenchymal cells	siRNA specific to oncogenic KrasG12D	Animals models	NA	The engineered exosomes suppressed cancer growth and significantly improved overall survival	(145)
Exosomes from HEK293T cells	Inhibitor of interaction between CD47 and SIRPα	Animals models	NA	promoting an intensive T cell infiltration and inhibiting tumor growth	(146)
Exosomes from tumor cells	Containing tumor antigens and immunostimulatory CpG DNA	Animals models	NA	Enhancing tumor antigen presentation capacity and antitumor effects of DCs	(147)
Exosomes from myeloma cell	Containing HSP70 and P1A tumor antigen	Animals models	NA	Promoting CTL responses and antitumour immunity	(148)
Exosomes from DCs	Delivering antigen and costimulatory molecules	Animals models	NA	Stimulating cytotoxic T lymphocytes and inducing efficient antitumor immunity	(149)
Exosomes from DCs	Delivering antigen and costimulatory molecules	IIIb and IV non-small cell lung cancer	I	Activating immune effectors and having long term stability of disease	(150)
EVs from CD8^+^ T cell	Targeting lesional mesenchymal cell	Animals models	NA	Preventing tumor progressions	(151)
Exosomes from NK cells	Expressing FasL and perforin	In vitro experiment	NA	Exerting a cytotoxic activity against target cells	(152)
Exosomes from M1-macrophages	Delivering proinflammatory signal to produce more Th1 cytokines	Animals models	NA	Inducing a stronger antigen-specific cytotoxic T cell response	(153)

### Immune checkpoint inhibitors

Immune checkpoint proteins are molecules that produce costimulatory or inhibitory signals in the immune response and regulate the host immune response under normal conditions. Recent studies have mainly focused on the immune checkpoint PD-1, its ligand PD-L1 and cytotoxic T lymphocyte-associated protein 4 (CTLA-4) which negatively regulate T cell function (154). PD⁃L1 highly expressed in tumors binds to PD-1 on the surface of T cells and limits T cell activation, resulting in tumor immune escape. Therefore, the application of PD-1 or PD-L1 antibody may restore the state of T cells and rectify the immunosuppression of TME for PD-L1 positive tumors. CTLA-4 is an immune checkpoint molecule expressed on Treg. Clearing Treg cells by anti CTLA-4 monoclonal antibodies relieves the inhibition of CTLs and activate T cell immune response. Combinations of immune checkpoint inhibitors with radiotherapy and/or chemotherapy have shown efficacy in pancreatic cancer. Ipilimumab, an anti-CTLA-4 IgG1 monoclonal antibody (mAb), is approved for cancer therapy in USA and Europe in 2011. A phase Ib clinical trial showed that gemcitabine and ipilimumab is a safe regimen with tolerable adverse events for pancreatic cancer, with a median progression-free survival (PFS) of 2.78 months (95% CI 1.61–4.83) and median overall survival (OS) of 6.90 months (95% CI 2.63–9.57) (123). In 2014, Japan and the United States approved pembrolizumab, the first anti-PD-1 monoclonal antibody, for metastatic melanoma. A phase I/II study (NCT02331251) confirmed the safety of pembrolizumab. Moreover, the study also demonstrated that the median PFS and OS with 9.1 and 15.0 months were achieved respectively by combining pembrolizumab with gemcitabine and nab-paclitaxel chemotherapy. The prognosis was slightly improved over previously reported results for standard regiments (124). The efficacy of tremelimumab, another anti-CTLA-4 inhibitor, was evaluated in a phase I trial (NCT00556023). The median OS was 7.4 months (95% CI 5.8-9.4 months) by combing tremelimumab with gemcitabine. Two patients achieved partial response and 7 showed stable disease for more than 10 weeks at the end of treatment (125). However, the efficacy of tremelimumab as monotherapy assessed by a phase II open label study (NCT02527434) was unsatisfactory with 18 out of 20 pancreatic cancer patients demonstrating progressive disease and a poor median OS of 4 months (95% CI 2.83–5.42) (126). The indications of immune checkpoint inhibitors are gradually extending, and there are also survival benefits in pancreatic cancer, renal cell carcinoma, gastric cancer, and non-small cell lung cancer. Blocking of the above two immune checkpoint proteins (PD-1/CTLA-4) may produces a synergistic effect on tumor. However, related complications such as autoimmune hepatitis, myocarditis, nervous system inflammation and pneumonia have been reported with the wide use of immune checkpoint inhibitors (155). It is reported that the mortality of autoimmune myocarditis caused by anti PD-1 therapy is as high as 46% (156). Therefore, effective intervention or method should be taken to avoid possible serious complications before the application of immune checkpoint drugs in clinical practice.

### Targeting extracellular matrix

Although the ECM in TME is not as important as infiltrating immune cells in tumor immunotherapy, this component has a critical impact on tumor immunotherapy. Methods have been adopted to reduce extracellular matrix hardness and fibrosis, thus improving immune cell infiltration and drug delivery (157). Inhibition of protein tyrosine kinase that is involved in matrix fiber formation can prolong the survival of pancreatic cancer and make them more sensitive to T cell adoptive reinfusion and PD-1 blocking therapy. The infiltration of immune cell into TME is enhanced *via* digesting the fibrous stroma through applying of IL-15 activated NK cells, whole-cell vaccines secreting GM-CSF and CD40 specific monoclonal antibody. TGF-β secreted by CAFs in pancreatic TME accelerates formation of fibrous matrix by transforming the phenotype of fibroblasts. Furthermore, TGF-β increases production of matrix proteins, including collagen, fibronectin, proteoglycans and tenascin *via* enhancing the expression of ECM-associated genes in epithelial cells, including collagen type 1 α1 (COL1A1), lysyl oxidase homologue 4 (LOXL4) and MMP (158). TGF-β can be used as a target for enhanced cancer chemotherapy and immunotherapy. The TβRI kinase inhibitor galunisertib (LY2157299) combining with gemcitabine improve the OS and PFS for patients with unresectable pancreatic cancer in a clinical trial (127). A multinational, phase Ib study (NCT02734160) evaluated the safety and efficacy of the galunisertib co-administered with the anti-programmed death-ligand 1 (PD-L1) antibody durvalumab in metastatic pancreatic cancer. Galunisertib in combination with durvalumab achieved a disease control rate of 25.0% and a confirmed objective response rate of 3.1%. Median OS and PFS were 5.72 months (95% CI: 4.01 to 8.38) and 1.87 months (95% CI: 1.58 to 3.09), respectively (128). The effect of nanomedicine characterized with high permeability, tumor-targeting and substantial retention function on extracellular matrix of pancreatic cancer has been confirmed. Pegylated recombinant human hyaluronidase (PECPH20) decrease intratumoural interstitial fluid pressure and increase in vessel diameter in the tumor-bearing mouse models. Preclinical studies have shown that PECPH20 combined with chemotherapeutic drugs substantially decrease tumor volume and improve the overall survival time (129). A clinical trial evaluated the efficacy of PECPH20 combined with modified FOLFIRINOX in patients with untreated metastatic pancreatic cancer. Compared with the modified FOLFIRINOX alone, the OS of patients in the combination group was shortened and the incidence of related adverse events was significantly increased. This combination resulted in decreased treatment duration due to increased toxicity (130). OS and PFS were not improved in patients with metastatic pancreatic cancer treated with PECPH20 combined with paclitaxel and gemcitabine (131). The disappointing results indicate that it is not enough to only target fibrous tissue proliferation. In addition, although ECM in pancreatic TME is a physical barrier that hinders drug delivery, it also plays a protective role in inhibiting tumor growth and progression. The strategy selectively targeting ECM barrier of pancreatic cancer is worth further exploring.

### Targeting stromal cells and cytokines in TME

In view of the inhibitory effect of MDSCs in tumor immunity, many studies focus on finding ways to block the signal transduction of MDSCs. Animal model studies have confirmed that gemcitabine significantly reduce MDSCs in the spleen without altering the number of T and B lymphocytes. Bruton tyrosine kinase (BTK), a non-receptor enzyme in the Tec kinase family, is critical to maintain the desmoplastic microenvironment surrounded by an abundance of Tregs, MDSCs, TAMs and MCs in pancreatic cancer. BTK inhibition converts M2-like macrophages to an M1-like phenotype and promotes the cytotoxicity of CD8^+^ T cells. Inhibition of BTK with ibrutinib enhances T cell-dependent antitumor immune responses and improves responsiveness of pancreas cancer to gemcitabine (159). A randomized phase II clinical trial (NCT02362048) reported that the combination of BTK inhibitor acalabrutinib and pembrolizumab led to reduction of reductions in granulocytic (CD15^+^) MDSCs in peripheral blood of patient with pancreatic cancer, but the overall response rate and disease control rate were disappointing (132). Many studies have confirmed the importance of CAFs in promoting the occurrence and development of pancreatic cancer. However, some problems exist in targeting CAFs. One is that CAFs are highly heterogeneous. Inflammatory CAFs with low expression of a-SMA and high expression of IL-6 promote tumor growth, while myofibroblastic CAFs with high expression of a-SMA inhibit tumor growth (160). In addition, the phenotype CAFs of different subtypes can be transformed intercellularly. Meflin-positive CAFs have tumor suppressive properties by remodeling the extracellular matrix. However, meflin-positive CAFs differentiate into cancer-promoting phenotypes with the tumor progresses (34). Therefore, how to identify and remodel various properties of CAFs is the key to target CAFs for pancreatic cancer treatment.

The safety and efficacy of DC-based immunotherapy for pancreatic cancer have been evaluated. Studies show that patients with pancreatic cancer receiving DC vaccination-based immunotherapy achieves a significantly better survival period (161, 162). Peptide-loaded DC vaccines selectively targeting tumor-associated antigens (TAAs) such as mucin 1 (MUC1), Wilms’ tumor gene 1 (WT1) and mutant K-RAS also enhance more cytotoxic lymphocyte response. Clinical trials showed that MUC1 peptide-loaded DCs were safe and effective for unresectable, recurrent or refractory pancreatic cancer (136–138). A phase I trial to investigated the safety and efficacy following treatment with DCs pulsed with a mixture of three types of WT1 peptides in combination with chemotherapy in pancreatic cancer and intrahepatic cholangiocarcinoma. The DC/WT1-I/II combined with chemotherapy effectively activated WT1-specific immune responses and promoted disease stability in advanced pancreatic cancer (139). DCs enhance more effectively the anti-tumor immune response when cultured in presence of toll-like receptor (TLR)-3 agonist poly-ICLC. Mehrotra, et al. (NCT01410968) showed that the addition of poly-ICLC to peptide-pulsed DCs was safe and produced increased numbers of tumor specific T cell population in patients with advanced pancreatic cancer (140). Even though DC vaccines-based immunotherapy have effective therapeutic effects on pancreatic cancer, the effect is limited because decreased expression of MHC molecules and costimulatory molecules on cancer cells mediates the tumor immune evasion. A series of clinical studies have shown that adoptive NK cell transfer has anti-tumor effects on pancreatic cancer. A clinical trial (NCT02718859) showed combining allogeneic NK cells with irreversible electroporation (IRE) had a synergistic effect on pancreatic cancer, demonstrating satisfactory short-term outcome and the quality of life of the patients (141). In addition, percutaneous IRE in combining with allogeneic NK cells also significantly extended the PFS and OS in advanced pancreatic cancer (142). Lenalidomide, initially approved by the U. S. Food and Drug Administration (FDA) for multiple myeloma (MM), has anti-tumor effects by augmenting the cytotoxicity of NK cells. A clinical trial (NCT01547260) found lenalidomide augmented a significant increase in the numbers of CD4^+^ and CD8^+^ T cells in patients with advanced pancreatic cancer. However, combination of lenalidomide with gemcitabine have no therapeutic impact compared to gemcitabine alone (143).

The tryptophan catabolic enzyme IDO produced by tumor cells in TME promote the activation of Treg cells and increase the infiltration of MDSCs. Therefore, depletion of IDO inhibits tumor stem cells proliferation pathways and improves immunotherapeutic vaccines susceptibility by reducing TLRs 2 to 9, NF-κβ1-2, Wnt/β-catenin and TGF-β (163). CD47 is expressed in a variety of tumor cells, and mediates a “don’t eat me” signal after binding the immune checkpoint protein SIRP-α expressed on myeloid cells (monocytes, macrophages, and DCs), which contributes to the resistance of tumors to phagocyte-dependent clearance. KWAR23, a SIRP α monoclonal antibody which blocks its binding to CD47, enhances myeloid cell-dependent tumor killing ability, activates neutrophils and macrophages, and inhibits tumor growth (144). IFN enhances function of DCs and NK cells, and improve the survival of T cells, which is already used in cancer therapy. Karakhanova et al. reported that INF-2α increased amount of IFN-γ and IL-10 in the serum, and promoted the DCs and NK cells activation following the IFN-2α therapy in pancreatic cancer patients. However, activation of anti-tumor immunity as well as immunosuppressive response are induced in the unspecific immunotherapy with INF-2α (133). IL-6, a glycoprotein of 184 amino acids, acts on myeloid progenitor cells and B and T lymphocytes. A phase I clinical study showed that IL-6 induced C-reactive protein and IgE levels, and had a suppressive effect on NK and lymphokine-activated killer activity in patients with advanced pancreatic cancer and colon cancer (134). Combined CXCR4 and PD-1 blockade may expand the benefit of chemotherapy. A phase IIa clinical trial (NCT02826486) conformed the synergistic effects of combing CXCR4 antagonist BL-8040 (motixafortide) with pembrolizumab and chemotherapy on metastatic pancreatic cancer (135).

### Extracellular vesicles

Accumulating evidence indicates that extracellular vesicles (EVs) including microvesicles (MVs) and exosomes mediate reciprocal communication between pancreatic cancer and stromal cells in TME, and ultimately exerts influence on the biologic features of recipient cells, thus promoting cancer progression and evasion of immune surveillance (164, 165). Exosomes derived from pancreatic cancer cause an imbalance of immune cells *via* increasing MDSCs, while reducing DCs. Tumor-derived EVs inhibit NK cell cytotoxicity by decreasing the expression of NKG2D, INF-γ and TNF-α (166). TGF-β1 contained in pancreatic cancer-derived EVs suppresses NK cell cytotoxic activity against tumor cells *via* SMAD2/3-dependent signal pathway, resulting in immune tolerance and tumor immunosuppression (167). The EVs are modified for clinical applications through artificially integration of specific loadings such as drugs or tumor targeting molecules. Exosomes from normal fibroblast-like mesenchymal cells carry siRNA specific to oncogenic KrasG12D which is commonly mutated in pancreatic cancer. The engineered exosomes suppressed cancer growth and significantly improved overall survival (145). Exosomes secreted from immune cells including DCs, macrophages and CD8^+^ T cells express SIRPα, PD1, or tumor antigen peptides, which can be used as immune modulators. SIRPa-exosomes, acting as immune checkpoint blockade, antagonizes the crosstalk between CD47 and SIRPa, and induce tumor phagocytosis. SIRPa-exosomes enhance effective anti-tumor T cell response and increase T cell infiltration in cancer (146). Engineered exosomes from tumor cells containing immunostimulatory CpG DNA or tumor antigens (TRP2, gp100, endogenous P1A tumor antigen and HSP70) stimulate DCs maturation and T cell immune responses (147, 148). EVs released from immune cells also can be used as a vaccine adjuvant in cancer immunotherapy. Clinical trials showed that exosomes from DCs loaded with tumor antigen activated the strong cytotoxicity of CD8^+^ T cells and promoted anti­tumor immune responses (149, 150). EVs derived from activated CD8^+^ T cell inhibit tumor progression by depletion of mesenchymal tumor stromal cells (151). Exosomes from NK cells and M1-like macrophages exert a stronger cytotoxic T cell response against tumor cells (152, 153). However, some challenges in EVs-based immunotherapy still remain including lack of methods for efficient isolation, purification, and identification of specific EV populations. Additionally, the antigen loading efficiency of EVs should be improved (168).

### Gene mutation and potential targets

Oncogenic K-Ras is involved in tumorigenesis in pancreatic cancer and contribute to immunosuppressive microenvironment. More than 85% patients with pancreatic cancers harbor the G12 mutation in K-Ras. K-Ras mutation increases robustly MEK/ERK activity by upregulating the EGFR and RAS levels (169). Due to the multiple alternative pathways of K-Ras, there is none of specific K-Ras inhibitors that have been applied in clinical practice. Moreover, inhibitors of downstream molecules of K-Ras such as RAF/MEK/ERK pathway or the PI3K/PDK1/AKT/mTOR pathway show little efficacy (170). Multidrug combinations of MEK inhibitors and PI3K inhibitors contribute to apoptosis in pancreatic cancer and achieve a longer median survival (171). Ulixertinib, an ERK inhibitor, combined with MEK inhibitors produces potent synergistic effects on tumor (172). More studies are needed in the evaluation of combinatorial effect of these therapies altogether to improve survival rate.

## Challenges and prospects

Immunotherapy has a positive therapeutic effect on patients with advanced pancreatic cancer. Tumors in TME dynamically evolves by blocking the effect of immunotherapy and increasing drug resistance. Because of the complexity of tumor immunosuppressive microenvironment, immunotherapy including immune checkpoint inhibitors, interfering with stromal cells or cytokines in pancreatic TME and cancer vaccines still need to be improved. The efficacy of immunotherapy combinations with chemoradiotherapy or other molecularly targeted agents is also disappointing for the majority of clinical trials. The main reasons hindering the effect of immunotherapy include: (1) the low response of host immune system to tumor antigen; (2) low infiltration of immune cells in pancreatic TME; (3) formation of immunosuppressive TME which is characterized by a profoundly desmoplastic stroma. A large number of immunosuppressive cells including MDSCs, TAMs, CAFs, and Tregs in pancreatic TME decrease the efficacy of immunotherapy (173–175). A variety of immunotherapeutic drugs targeting TME have been developed. However, it is evident that single-agent immunotherapies are unlikely to be successful in pancreatic cancer due to the diversified immunosuppressive signals, and the heterogeneity of TME at different stages of cancer progression is complex, which limits the accuracy and effectiveness of immunotherapy. Considering the limitations of current immunotherapy strategy, it may be improved from the following aspects: (1) screening markers sensitive to the immunotherapy strategies. Sensitive biomarkers predict the effectiveness of immunotherapy, dynamically monitor the treatment process, and adjust the therapeutic schedule accordingly; (2) combine multiple immunotherapy strategies. It is necessary to explore the combined treatment strategies that strengthen the effect of cytotoxicity and improve the sensitivity of immunotherapy through the combination of radiotherapy, chemotherapy and immunotherapy, or the application of multiple immunotherapy strategies; (3) develop new immune activation strategies. By developing a new immune activation vector will affect the immune state of pancreatic TME, and enhance immune response against tumor (176). Therefore, it is still necessary to explore the underlying mechanism of immunosuppressive TME, which contribute to the success of combination therapy for pancreatic cancer.

## Author contributions

SL and YL designed the review structure and wrote the manuscript. SX performed the scientific literature search, collected and analyzed data. WP, JW and HZ prepared the tables and figures. All authors contributed to the article and approved the submitted version.

## References

[1] Siegel RL Miller KD Fuchs HE Jemal A. Cancer statistics, 2021. CA Cancer J Clin (2021) 71(1):7–33. doi: 10.3322/caac.21654 33433946

[2] Grossberg AJ Chu LC Deig CR Fishman EK Hwang WL Maitra A. Multidisciplinary standards of care and recent progress in pancreatic ductal adenocarcinoma. CA Cancer J Clin (2020) 70(5):375–403. doi: 10.3322/caac.21626 32683683 PMC7722002

[3] Farhood B Najafi M Mortezaee K. CD8+ cytotoxic T lymphocytes in cancer immunotherapy: A review. J Cell Physiol (2019) 234(6):8509–21. doi: 10.1002/jcp.27782 30520029

[4] Ho WJ Jaffee EM Zheng L. The tumour microenvironment in pancreatic cancer - clinical challenges and opportunities. Nat Rev Clin Oncol (2020) 17(9):527–40. doi: 10.1038/s41571-020-0363-5 PMC7442729 32398706

[5] Mao X Xu J Wang W Liang C Hua J Liu J. Crosstalk between cancer-associated fibroblasts and immune cells in the tumor microenvironment: new findings and future perspectives. Mol Cancer (2021) 20(1):131. doi: 10.1186/s12943-021-01428-1 34635121 PMC8504100

[6] Osipov A Saung MT Zheng L Murphy AG. Small molecule immunomodulation: the tumor microenvironment and overcoming immune escape. J Immunother Cancer (2019) 7(1):224. doi: 10.1186/s40425-019-0667-0 31439034 PMC6704558

[7] Huber M Brehm CU Gress TM Buchholz M Alashkar Alhamwe B von Strandmann EP. The immune microenvironment in pancreatic cancer. Int J Mol Sci (2020) 21(19):7307. doi: 10.3390/ijms21197307 33022971 PMC7583843

[8] Dougan SK. The pancreatic cancer microenvironment. Cancer J (2017) 23(6):321–5. doi: 10.1097/PPO.0000000000000288 29189327

[9] Peran I Dakshanamurthy S McCoy MD Mavropoulos A Allo B Sebastian A. Cadherin 11 promotes immunosuppression and extracellular matrix deposition to support growth of pancreatic tumors and resistance to gemcitabine in mice. Gastroenterology (2021) 160(4):1359–72.e13. doi: 10.1053/j.gastro.2020.11.044 33307028 PMC7956114

[10] Ren B Cui M Yang G Wang H Feng M You L. Tumor microenvironment participates in metastasis of pancreatic cancer. Mol Cancer (2018) 17(1):108. doi: 10.1186/s12943-018-0858-1 30060755 PMC6065152

[11] Nakamura K Smyth MJ. Myeloid immunosuppression and immune checkpoints in the tumor microenvironment. Cell Mol Immunol (2020) 17(1):1–12. doi: 10.1038/s41423-019-0306-1 31611651 PMC6952382

[12] Condamine T Mastio J Gabrilovich DI. Transcriptional regulation of myeloid-derived suppressor cells. J Leukoc Biol (2015) 98(6):913–22. doi: 10.1189/jlb.4RI0515-204R PMC4661041 26337512

[13] Gabrilovich DI. Myeloid-derived suppressor cells. Cancer Immunol Res (2017) 5(1):3–8. doi: 10.1158/2326-6066.CIR-16-0297 28052991 PMC5426480

[14] Mace TA Ameen Z Collins A Wojcik S Mair M Young GS. Pancreatic cancer-associated stellate cells promote differentiation of myeloid-derived suppressor cells in a STAT3-dependent manner. Cancer Res (2013) 73(10):3007–18. doi: 10.1158/0008-5472.CAN-12-4601 PMC3785672 23514705

[15] Ostrand-Rosenberg S Fenselau C. Myeloid-derived suppressor cells: Immune-suppressive cells that impair antitumor immunity and are sculpted by their environment. J Immunol (2018) 200(2):422–31. doi: 10.4049/jimmunol.1701019 PMC5765878 29311384

[16] Kumar V Patel S Tcyganov E Gabrilovich DI. The nature of myeloid-derived suppressor cells in the tumor microenvironment. Trends Immunol (2016) 37(3):208–20. doi: 10.1016/j.it.2016.01.004 PMC4775398 26858199

[17] Lunardi S Muschel RJ Brunner TB. The stromal compartments in pancreatic cancer: are there any therapeutic targets? Cancer Lett (2014) 343(2):147–55. doi: 10.1016/j.canlet.2013.09.039 24141189

[18] Yan X Takahara M Xie L Gondo C Setsu N Oda Y. Arginine metabolism in soft tissue sarcoma. J Dermatol Sci (2011) 61(3):211–5. doi: 10.1016/j.jdermsci.2010.12.009 21292446

[19] Zea AH Rodriguez PC Culotta KS Hernandez CP DeSalvo J Ochoa JB. L-arginine modulates CD3zeta expression and T cell function in activated human T lymphocytes. Cell Immunol (2004) 232(1-2):21–31. doi: 10.1016/j.cellimm.2005.01.004 15922712

[20] Ohl K Tenbrock K. Reactive oxygen species as regulators of MDSC-mediated immune suppression. Front Immunol (2018) 9:2499. doi: 10.3389/fimmu.2018.02499 30425715 PMC6218613

[21] Corzo CA Cotter MJ Cheng P Cheng F Kusmartsev S Sotomayor E. Mechanism regulating reactive oxygen species in tumor-induced myeloid-derived suppressor cells. J Immunol (2009) 182(9):5693–701. doi: 10.4049/jimmunol.0900092 PMC2833019 19380816

[22] Schmielau J Finn OJ. Activated granulocytes and granulocyte-derived hydrogen peroxide are the underlying mechanism of suppression of t-cell function in advanced cancer patients. Cancer Res (2001) 61(12):4756–60. 11406548

[23] Wei J Zhang M Zhou J. Myeloid-derived suppressor cells in major depression patients suppress T-cell responses through the production of reactive oxygen species. Psychiatry Res (2015) 228(3):695–701. doi: 10.1016/j.psychres.2015.06.002 26165964

[24] Lelis FJN Jaufmann J Singh A Fromm K Teschner AC Pöschel S. Myeloid-derived suppressor cells modulate b-cell responses. Immunol Lett (2017) 188:108–15. doi: 10.1016/j.imlet.2017.07.003 28687234

[25] Grzywa TM Sosnowska A Matryba P Rydzynska Z Jasinski M Nowis D. Myeloid cell-derived arginase in cancer immune response. Front Immunol (2020) 11:938. doi: 10.3389/fimmu.2020.00938 32499785 PMC7242730

[26] Malek E de Lima M Letterio JJ Kim BG Finke JH Driscoll JJ. Myeloid-derived suppressor cells: The green light for myeloma immune escape. Blood Rev (2016) 30(5):341–8. doi: 10.1016/j.blre.2016.04.002 PMC6411302 27132116

[27] Pan PY Ma G Weber KJ Ozao-Choy J Wang G Yin B. Immune stimulatory receptor CD40 is required for T-cell suppression and T regulatory cell activation mediated by myeloid-derived suppressor cells in cancer. Cancer Res (2010) 70(1):99–108. doi: 10.1158/0008-5472.CAN-09-1882 19996287 PMC2805053

[28] Huang B Pan PY Li Q Sato AI Levy DE Bromberg J. Gr-1+CD115+ immature myeloid suppressor cells mediate the development of tumor-induced T regulatory cells and T-cell anergy in tumor-bearing host. Cancer Res (2006) 66(2):1123–31. doi: 10.1158/0008-5472.CAN-05-1299 16424049

[29] Li T Liu T Zhu W Xie S Zhao Z Feng B. Targeting MDSC for immune-checkpoint blockade in cancer immunotherapy: Current progress and new prospects. Clin Med Insights Oncol (2021) 15:11795549211035540. doi: 10.1177/11795549211035540 34408525 PMC8365012

[30] Noman MZ Desantis G Janji B Hasmim M Karray S Dessen P. PD-L1 is a novel direct target of HIF-1α, and its blockade under hypoxia enhanced MDSC-mediated T cell activation. J Exp Med (2014) 211(5):781–90. doi: 10.1084/jem.20131916 PMC4010891 24778419

[31] Li Y Wang J Wang H Zhang S Wei Y Liu S. The interplay between inflammation and stromal components in pancreatic cancer. Front Immunol (2022) 13:850093. doi: 10.3389/fimmu.2022.850093 35493517 PMC9046560

[32] Li L Yu R Cai T Chen Z Lan M Zou T. Effects of immune cells and cytokines on inflammation and immunosuppression in the tumor microenvironment. Int Immunopharmacol (2020) 88:106939. doi: 10.1016/j.intimp.2020.106939 33182039

[33] Chen J Ye Y Liu P Yu W Wei F Li H. Suppression of T cells by myeloid-derived suppressor cells in cancer. Hum Immunol (2017) 78(2):113–9. doi: 10.1016/j.humimm.2016.12.001 27939507

[34] Geng X Chen H Zhao L Hu J Yang W Li G. Cancer-associated fibroblast (CAF) heterogeneity and targeting therapy of CAFs in pancreatic cancer. Front Cell Dev Biol (2021) 9:655152. doi: 10.3389/fcell.2021.655152 34336821 PMC8319605

[35] Mhaidly R Mechta-Grigoriou F. Fibroblast heterogeneity in tumor micro-environment: Role in immunosuppression and new therapies. Semin Immunol (2020) 48:101417. doi: 10.1016/j.smim.2020.101417 33077325

[36] De Jaeghere EA Denys HG De Wever O. Fibroblasts fuel immune escape in the tumor microenvironment. Trends Cancer (2019) 5(11):704–23. doi: 10.1016/j.trecan.2019.09.009 31735289

[37] Cho H Seo Y Loke KM Kim SW Oh SM Kim JH. Cancer-stimulated CAFs enhance monocyte differentiation and protumoral TAM activation *via* IL6 and GM-CSF secretion. Clin Cancer Res (2018) 24(21):5407–21. doi: 10.1158/1078-0432.CCR-18-0125 29959142

[38] Deng Y Cheng J Fu B Liu W Chen G Zhang Q. Hepatic carcinoma-associated fibroblasts enhance immune suppression by facilitating the generation of myeloid-derived suppressor cells. Oncogene (2017) 36(8):1090–101. doi: 10.1038/onc.2016.273 27593937

[39] Xiang H Ramil CP Hai J Zhang C Wang H Watkins AA. Cancer-associated fibroblasts promote immunosuppression by inducing ROS-generating monocytic MDSCs in lung squamous cell carcinoma. Cancer Immunol Res (2020) 8(4):436–50. doi: 10.1158/2326-6066.CIR-19-0507 32075803

[40] Kato T Noma K Ohara T Kashima H Katsura Y Sato H. Cancer-associated fibroblasts affect intratumoral CD8+ and FoxP3+ T cells *Via* IL6 in the tumor microenvironment. Clin Cancer Res (2018) 24(19):4820–33. doi: 10.1158/1078-0432.CCR-18-0205 29921731

[41] Donatelli SS Zhou JM Gilvary DL Eksioglu EA Chen X Cress WD. TGF-β-inducible microRNA-183 silences tumor-associated natural killer cells. Proc Natl Acad Sci USA (2014) 111(11):4203–8. doi: 10.1073/pnas.1319269111 PMC3964044 24586048

[42] Balsamo M Scordamaglia F Pietra G Manzini C Cantoni C Boitano M. Melanoma-associated fibroblasts modulate NK cell phenotype and antitumor cytotoxicity. Proc Natl Acad Sci USA (2009) 106(49):20847–52. doi: 10.1073/pnas.0906481106 PMC2791633 19934056

[43] Feig C Jones JO Kraman M Wells RJ Deonarine A Chan DS. Targeting CXCL12 from FAP-expressing carcinoma-associated fibroblasts synergizes with anti-PD-L1 immunotherapy in pancreatic cancer. Proc Natl Acad Sci USA (2013) 110(50):20212–7. doi: 10.1073/pnas.1320318110 PMC3864274 24277834

[44] Wu F Yang J Liu J Wang Y Mu J Zeng Q. Signaling pathways in cancer-associated fibroblasts and targeted therapy for cancer. Signal Transduct Target Ther (2021) 6(1):218. doi: 10.1038/s41392-021-00641-0 34108441 PMC8190181

[45] Seifert AM Eymer A Heiduk M Wehner R Tunger A von Renesse J. PD-1 expression by lymph node and intratumoral regulatory T cells is associated with lymph node metastasis in pancreatic cancer. Cancers (Basel) (2020) 12(10):2756. doi: 10.3390/cancers12102756 32987956 PMC7599971

[46] Whiteside TL. FOXP3+ treg as a therapeutic target for promoting anti-tumor immunity. Expert Opin Ther Targets (2018) 22(4):353–63. doi: 10.1080/14728222.2018.1451514 PMC6126897 29532697

[47] Lunardi S Lim SY Muschel RJ Brunner TB. IP-10/CXCL10 attracts regulatory T cells: Implication for pancreatic cancer. Oncoimmunology (2015) 4(9):e1027473. doi: 10.1080/2162402X.2015.1027473 26405599 PMC4570127

[48] Wang X Wang L Mo Q Dong Y Wang G Ji A. Changes of Th17/Treg cell and related cytokines in pancreatic cancer patients. Int J Clin Exp Pathol (2015) 8(5):5702–8. PMC4503155 26191284

[49] Nummer D Suri-Payer E Schmitz-Winnenthal H Bonertz A Galindo L Antolovich D. Role of tumor endothelium in CD4+ CD25+ regulatory T cell infiltration of human pancreatic carcinoma. J Natl Cancer Inst (2007) 99(15):1188–99. doi: 10.1093/jnci/djm064 17652277

[50] Bommireddy R Doetschman T. TGFbeta1 and treg cells: alliance for tolerance. Trends Mol Med (2007) 13(11):492–501. doi: 10.1016/j.molmed.2007.08.005 17977791 PMC2805009

[51] Kryczek I Wei S Zou L Zhu G Mottram P Xu H. Cutting edge: induction of B7-H4 on APCs through IL-10: novel suppressive mode for regulatory T cells. J Immunol (2006) 177(1):40–4. doi: 10.4049/jimmunol.177.1.40 16785496

[52] Nakamura T Shima T Saeki A Hidaka T Nakashima A Takikawa O. Expression of indoleamine 2, 3-dioxygenase and the recruitment of Foxp3-expressing regulatory T cells in the development and progression of uterine cervical cancer. Cancer Sci (2007) 98(6):874–81. doi: 10.1111/j.1349-7006.2007.00470.x PMC11159745 17433037

[53] Cassetta L Pollard JW. Tumor-associated macrophages. Curr Biol (2020) 30(6):R246–8. doi: 10.1016/j.cub.2020.01.031 32208142

[54] Medvedeva GF Kuzmina DO Nuzhina J Shtil AA Dukhinova MS. How macrophages become transcriptionally dysregulated: A hidden impact of antitumor therapy. Int J Mol Sci (2021) 22(5):2662. doi: 10.3390/ijms22052662 33800829 PMC7961970

[55] Pan Y Yu Y Wang X Zhang T. Tumor-associated macrophages in tumor immunity. Front Immunol (2020) 11:583084. doi: 10.3389/fimmu.2020.583084 33365025 PMC7751482

[56] Piao C Zhang WM Li TT Zhang CC Qiu S Liu Y. Complement 5a stimulates macrophage polarization and contributes to tumor metastases of colon cancer. Exp Cell Res (2018) 366(2):127–38. doi: 10.1016/j.yexcr.2018.03.009 29551360

[57] Dong L Chen C Zhang Y Guo P Wang Z Li J. The loss of RNA N6-adenosine methyltransferase Mettl14 in tumor-associated macrophages promotes CD8+ T cell dysfunction and tumor growth. Cancer Cell (2021) 39(7):945–57.e10. doi: 10.1016/j.ccell.2021.04.016 34019807

[58] Mazzone M Menga A Castegna A. Metabolism and TAM functions-it takes two to tango. FEBS J (2018) 285(4):700–16. doi: 10.1111/febs.14295 29055087

[59] Yan S Wan G. Tumor-associated macrophages in immunotherapy. FEBS J (2021) 288(21):6174–86. doi: 10.1111/febs.15726 33492779

[60] Rahma OE Hodi FS. The intersection between tumor angiogenesis and immune suppression. Clin Cancer Res (2019) 25(18):5449–57. doi: 10.1158/1078-0432.CCR-18-1543 30944124

[61] Zhou J Li X Wu X Zhang T Zhu Q Wang X. Exosomes released from tumor-associated macrophages transfer miRNAs that induce a Treg/Th17 cell imbalance in epithelial ovarian cancer. Cancer Immunol Res (2018) 6(12):1578–92. doi: 10.1158/2326-6066.CIR-17-0479 30396909

[62] Podojil JR Miller SD. Potential targeting of B7-H4 for the treatment of cancer. Immunol Rev (2017) 276(1):40–51. doi: 10.1111/imr.12530 28258701 PMC5630270

[63] Zhang M Huang L Ding G Huang H Cao G Sun X. Interferon gamma inhibits CXCL8-CXCR2 axis mediated tumor-associated macrophages tumor trafficking and enhances anti-PD1 efficacy in pancreatic cancer. J Immunother Cancer (2020) 8(1):e000308. doi: 10.1136/jitc-2019-000308 32051287 PMC7057481

[64] Schnittert J Bansal R Prakash J. Targeting pancreatic stellate cells in cancer. Trends Cancer (2019) 5(2):128–42. doi: 10.1016/j.trecan.2019.01.001 30755305

[65] Farran B Nagaraju GP. The dynamic interactions between the stroma, pancreatic stellate cells and pancreatic tumor development: Novel therapeutic targets. Cytokine Growth Factor Rev (2019) 48:11–23. doi: 10.1016/j.cytogfr.2019.07.001 31331827

[66] Hu F Lou N Jiao J Guo F Xiang H Shang D. Macrophages in pancreatitis: Mechanisms and therapeutic potential. BioMed Pharmacother (2020) 131:110693. doi: 10.1016/j.biopha.2020.110693 32882586

[67] Tang D Yuan Z Xue X Lu Z Zhang Y Wang H. High expression of galectin-1 in pancreatic stellate cells plays a role in the development and maintenance of an immunosuppressive microenvironment in pancreatic cancer. Int J Cancer (2012) 130(10):2337–48. doi: 10.1002/ijc.26290 21780106

[68] Masamune A Shimosegawa T. Pancreatic stellate cells: A dynamic player of the intercellular communication in pancreatic cancer. Clin Res Hepatol Gastroenterol (2015) 39 Suppl 1:S98–103. doi: 10.1016/j.clinre.2015.05.018 26189983

[69] Wu L Zhang XH. Tumor-associated neutrophils and macrophages-heterogenous but not chaotic. Front Immunol (2020) 11:553967. doi: 10.3389/fimmu.2020.553967 33343560 PMC7738476

[70] Fridlender ZG Sun J Kim S Kapoor V Cheng G Ling L. Polarization of tumor-associated neutrophil phenotype by TGF-beta: "N1" versus "N2" TAN. Cancer Cell (2009) 16(3):183–94. doi: 10.1016/j.ccr.2009.06.017 PMC2754404 19732719

[71] Zhou SL Zhou ZJ Hu ZQ Huang XW Wang Z Chen EB. Tumor-associated neutrophils recruit macrophages and T-regulatory cells to promote progression of hepatocellular carcinoma and resistance to sorafenib. Gastroenterology (2016) 150(7):1646–58.e17. doi: 10.1053/j.gastro.2016.02.040 26924089

[72] Keeley T Costanzo-Garvey DL Cook LM. Unmasking the many faces of tumor-associated neutrophils and macrophages: Considerations for targeting innate immune cells in cancer. Trends Cancer (2019) 5(12):789–98. doi: 10.1016/j.trecan.2019.10.013 31813456

[73] Sivori S Pende D Quatrini L Pietra G Della Chiesa M Vacca P. NK cells and ILCs in tumor immunotherapy. Mol Aspects Med (2021) 80:100870. doi: 10.1016/j.mam.2020.100870 32800530

[74] Bald T Krummel MF Smyth MJ Barry KC. The NK cell-cancer cycle: advances and new challenges in NK cell-based immunotherapies. Nat Immunol (2020) 21(8):835–47. doi: 10.1038/s41590-020-0728-z PMC8406687 32690952

[75] Wu SY Fu T Jiang YZ Shao ZM. Natural killer cells in cancer biology and therapy. Mol Cancer (2020) 19(1):120. doi: 10.1186/s12943-020-01238-x 32762681 PMC7409673

[76] Wu J Lanier LL. Natural killer cells and cancer. Adv Cancer Res (2003) 90:127–56. doi: 10.1016/s0065-230x(03)90004-2 14710949

[77] Zhou J Zhang S Guo C. Crosstalk between macrophages and natural killer cells in the tumor microenvironment. Int Immunopharmacol (2021) 101(Pt B):108374. doi: 10.1016/j.intimp.2021.108374 34824036

[78] Cooper MA Fehniger TA Fuchs A Colonna M Caligiuri MA. NK cell and DC interactions. Trends Immunol (2004) 25(1):47–52. doi: 10.1016/j.it.2003.10.012 14698284

[79] Jacobs B Ullrich E. The interaction of NK cells and dendritic cells in the tumor environment: how to enforce NK cell & DC action under immunosuppressive conditions? Curr Med Chem (2012) 19(12):1771–9. doi: 10.2174/092986712800099857 22414086

[80] Mahmood S Upreti D Sow I Amari A Nandagopal S Kung SK. Bidirectional interactions of NK cells and dendritic cells in immunotherapy: current and future perspective. Immunotherapy (2015) 7(3):301–8. doi: 10.2217/imt.14.122 25804481

[81] Aponte-López A Muñoz-Cruz S. Mast cells in the tumor microenvironment. Adv Exp Med Biol (2020) 1273:159–73. doi: 10.1007/978-3-030-49270-0_9 33119881

[82] Strouch MJ Cheon EC Salabat MR Krantz SB Gounaris E Melstrom LG. Crosstalk between mast cells and pancreatic cancer cells contributes to pancreatic tumor progression. Clin Cancer Res (2010) 16(8):2257–65. doi: 10.1158/1078-0432.CCR-09-1230 PMC3122919 20371681

[83] Ma Y Hwang RF Logsdon CD Ullrich SE. Dynamic mast cell-stromal cell interactions promote growth of pancreatic cancer. Cancer Res (2013) 73(13):3927–37. doi: 10.1158/0008-5472.CAN-12-4479 PMC3702652 23633481

[84] Porcelli L Iacobazzi RM Di Fonte R Serratì S Intini A Solimando AG. CAFs and TGF-β signaling activation by mast cells contribute to resistance to Gemcitabine/Nabpaclitaxel in pancreatic cancer. Cancers (Basel) (2019) 11(3):330. doi: 10.3390/cancers11030330 30866547 PMC6468868

[85] Khan MW Keshavarzian A Gounaris E Melson JE Cheon EC Blatner NR. PI3K/AKT signaling is essential for communication between tissue-infiltrating mast cells, macrophages, and epithelial cells in colitis-induced cancer. Clin Cancer Res (2013) 19(9):2342–54. doi: 10.1158/1078-0432.CCR-12-2623 PMC3947836 23487439

[86] Yang Z Zhang B Li D Lv M Huang C Shen GX. Mast cells mobilize myeloid-derived suppressor cells and treg cells in tumor microenvironment *via* IL-17 pathway in murine hepatocarcinoma model. PloS One (2010) 5(1):e8922. doi: 10.1371/journal.pone.0008922 20111717 PMC2811741

[87] Hegde S Leader AM Merad M. MDSC: Markers, development, states, and unaddressed complexity. Immunity (2021) 54(5):875–84. doi: 10.1016/j.immuni.2021.04.004 PMC8709560 33979585

[88] Bruger AM Dorhoi A Esendagli G Barczyk-Kahlert K van der Bruggen P Lipoldova M. How to measure the immunosuppressive activity of MDSC: assays, problems and potential solutions. Cancer Immunol Immunother (2019) 68(4):631–44. doi: 10.1007/s00262-018-2170-8 PMC11028070 29785656

[89] Costa A Kieffer Y Scholer-Dahirel A Pelon F Bourachot B Cardon M. Fibroblast heterogeneity and immunosuppressive environment in human breast cancer. Cancer Cell (2018) 33(3):463–79.e10. doi: 10.1016/j.ccell.2018.01.011 29455927

[90] Pelon F Bourachot B Kieffer Y Magagna I Mermet-Meillon F Bonnet I. Cancer-associated fibroblast heterogeneity in axillary lymph nodes drives metastases in breast cancer through complementary mechanisms. Nat Commun (2020) 11(1):404. doi: 10.1038/s41467-019-14134-w 31964880 PMC6972713

[91] Givel AM Kieffer Y Scholer-Dahirel A Sirven P Cardon M Pelon F. miR200-regulated CXCL12β promotes fibroblast heterogeneity and immunosuppression in ovarian cancers. Nat Commun (2018) 9(1):1056. doi: 10.1038/s41467-018-03348-z 29535360 PMC5849633

[92] Lakins MA Ghorani E Munir H Martins CP Shields JD. Cancer-associated fibroblasts induce antigen-specific deletion of CD8 + T cells to protect tumour cells. Nat Commun (2018) 9(1):948. doi: 10.1038/s41467-018-03347-0 29507342 PMC5838096

[93] Takahashi H Sakakura K Kawabata-Iwakawa R Rokudai S Toyoda M Nishiyama M. Immunosuppressive activity of cancer-associated fibroblasts in head and neck squamous cell carcinoma. Cancer Immunol Immunother (2015) 64(11):1407–17. doi: 10.1007/s00262-015-1742-0 PMC11029788 26201938

[94] Kieffer Y Hocine HR Gentric G Pelon F Bernard C Bourachot B. Single-cell analysis reveals fibroblast clusters linked to immunotherapy resistance in cancer. Cancer Discovery (2020) 10(9):1330–51. doi: 10.1158/2159-8290.CD-19-1384 32434947

[95] Li Z Zhou J Zhang J Li S Wang H Du J. Cancer-associated fibroblasts promote PD-L1 expression in mice cancer cells *via* secreting CXCL5. Int J Cancer (2019) 145(7):1946–57. doi: 10.1002/ijc.32278 PMC6767568 30873585

[96] Yang X Lin Y Shi Y Li B Liu W Yin W. FAP promotes immunosuppression by cancer-associated fibroblasts in the tumor microenvironment *via* STAT3-CCL2 signaling. Cancer Res (2016) 76(14):4124–35. doi: 10.1158/0008-5472.CAN-15-2973 27216177

[97] Bughda R Dimou P D'Souza RR Klampatsa A. Fibroblast activation protein (FAP)-targeted CAR-T cells: Launching an attack on tumor stroma. Immunotargets Ther (2021) 10:313–23. doi: 10.2147/ITT.S291767 PMC8354246 34386436

[98] Duperret EK Trautz A Ammons D Perales-Puchalt A Wise MC Yan J. Alteration of the tumor stroma using a consensus DNA vaccine targeting fibroblast activation protein (FAP) synergizes with antitumor vaccine therapy in mice. Clin Cancer Res (2018) 24(5):1190–201. doi: 10.1158/1078-0432.CCR-17-2033 PMC5844837 29269377

[99] Chauhan VP Chen IX Tong R Ng MR Martin JD Naxerova K. Reprogramming the microenvironment with tumor-selective angiotensin blockers enhances cancer immunotherapy. Proc Natl Acad Sci USA (2019) 116(22):10674–80. doi: 10.1073/pnas.1819889116 PMC6561160 31040208

[100] Gorchs L Ahmed S Mayer C Knauf A Fernández Moro C Svensson M. The vitamin d analogue calcipotriol promotes an anti-tumorigenic phenotype of human pancreatic CAFs but reduces T cell mediated immunity. Sci Rep (2020) 10(1):17444. doi: 10.1038/s41598-020-74368-3 33060625 PMC7562723

[101] Kamali Zonouzi S Pezeshki PS Razi S Rezaei N. Cancer-associated fibroblasts in colorectal cancer. Clin Transl Oncol (2022) 24(5):757–69. doi: 10.1007/s12094-021-02734-2 34839457

[102] Wu X Hu W Lu L Zhao Y Zhou Y Xiao Z. Repurposing vitamin d for treatment of human malignancies *via* targeting tumor microenvironment. Acta Pharm Sin B (2019) 9(2):203–19. doi: 10.1016/j.apsb.2018.09.002 PMC6437556 30972274

[103] Morita M Okuyama M Akutsu T Ohdaira H Suzuki Y Urashima M. Vitamin d supplementation regulates postoperative serum levels of PD-L1 in patients with digestive tract cancer and improves survivals in the highest quintile of PD-L1: A *Post hoc* analysis of the AMATERASU randomized controlled trial. Nutrients (2021) 13(6):1987. doi: 10.3390/nu13061987 34207794 PMC8228230

[104] Tanaka A Sakaguchi S. Regulatory T cells in cancer immunotherapy. Cell Res (2017) 27(1):109–18. doi: 10.1038/cr.2016.151 PMC5223231 27995907

[105] Zhang Y Lazarus J Steele NG Yan W Lee HJ Nwosu ZC. Regulatory T-cell depletion alters the tumor microenvironment and accelerates pancreatic carcinogenesis. Cancer Discovery (2020) 10(3):422–39. doi: 10.1158/2159-8290.CD-19-0958 PMC7224338 31911451

[106] Ohue Y Nishikawa H. Regulatory T (Treg) cells in cancer: Can treg cells be a new therapeutic target? Cancer Sci (2019) 110(7):2080–9. doi: 10.1111/cas.14069 PMC6609813 31102428

[107] Masucci MT Minopoli M Carriero MV. Tumor associated neutrophils. their role in tumorigenesis, metastasis, prognosis and therapy. Front Oncol (2019) 9:1146. doi: 10.3389/fonc.2019.01146 31799175 PMC6874146

[108] Gardner A Ruffell B. Dendritic cells and cancer immunity. Trends Immunol (2016) 37(12):855–65. doi: 10.1016/j.it.2016.09.006 PMC5135568 27793569

[109] Martinek J Wu TC Cadena D Banchereau J Palucka K. Interplay between dendritic cells and cancer cells. Int Rev Cell Mol Biol (2019) 348:179–215. doi: 10.1016/bs.ircmb.2019.07.008 31810553

[110] Zhou Y Slone N Chrisikos TT Kyrysyuk O Babcock RL Medik YB. Vaccine efficacy against primary and metastatic cancer with *in vitro*-generated CD103+ conventional dendritic cells. J Immunother Cancer (2020) 8(1):e000474. doi: 10.1136/jitc-2019-000474 32273347 PMC7254126

[111] Salmon H Idoyaga J Rahman A Leboeuf M Remark R Jordan S. Expansion and activation of CD103(+) dendritic cell progenitors at the tumor site enhances tumor responses to therapeutic PD-L1 and BRAF inhibition. Immunity (2016) 44(4):924–38. doi: 10.1016/j.immuni.2016.03.012 PMC4980762 27096321

[112] Chiba S Baghdadi M Akiba H Yoshiyama H Kinoshita I Dosaka-Akita H. Tumor-infiltrating DCs suppress nucleic acid-mediated innate immune responses through interactions between the receptor TIM-3 and the alarmin HMGB1. Nat Immunol (2012) 13(9):832–42. doi: 10.1038/ni.2376 PMC3622453 22842346

[113] Yin X Chen S Eisenbarth SC. Dendritic cell regulation of T helper cells. Annu Rev Immunol (2021) 39:759–90. doi: 10.1146/annurev-immunol-101819-025146 33710920

[114] Kenkel JA Tseng WW Davidson MG Tolentino LL Choi O Bhattacharya N. An immunosuppressive dendritic cell subset accumulates at secondary sites and promotes metastasis in pancreatic cancer. Cancer Res (2017) 77(15):4158–70. doi: 10.1158/0008-5472.CAN-16-2212 PMC5550516 28611041

[115] Wculek SK Cueto FJ Mujal AM Melero I Krummel MF Sancho D. Dendritic cells in cancer immunology and immunotherapy. Nat Rev Immunol (2020) 20(1):7–24. doi: 10.1038/s41577-019-0210-z 31467405

[116] Nava S Lisini D Frigerio S Bersano A. Dendritic cells and cancer immunotherapy: The adjuvant effect. Int J Mol Sci (2021) 22(22):12339. doi: 10.3390/ijms222212339 34830221 PMC8620771

[117] Yang J Eresen A Shangguan J Ma Q Yaghmai V Zhang Z. Irreversible electroporation ablation overcomes tumor-associated immunosuppression to improve the efficacy of DC vaccination in a mice model of pancreatic cancer. Oncoimmunology (2021) 10(1):1875638. doi: 10.1080/2162402X.2021.1875638 33643692 PMC7872063

[118] Bauer C Sterzik A Bauernfeind F Duewell P Conrad C Kiefl R. Concomitant gemcitabine therapy negatively affects DC vaccine-induced CD8(+) T-cell and b-cell responses but improves clinical efficacy in a murine pancreatic carcinoma model. Cancer Immunol Immunother (2014) 63(4):321–33. doi: 10.1007/s00262-013-1510-y PMC11029406 24384835

[119] Mayanagi S Kitago M Sakurai T Matsuda T Fujita T Higuchi H. Phase I pilot study of wilms tumor gene 1 peptide-pulsed dendritic cell vaccination combined with gemcitabine in pancreatic cancer. Cancer Sci (2015) 106(4):397–406. doi: 10.1111/cas.12621 25614082 PMC4409883

[120] Boullart AC Aarntzen EH Verdijk P Jacobs JF Schuurhuis DH Benitez-Ribas D. Maturation of monocyte-derived dendritic cells with toll-like receptor 3 and 7/8 ligands combined with prostaglandin E2 results in high interleukin-12 production and cell migration. Cancer Immunol Immunother (2008) 57(11):1589–97. doi: 10.1007/s00262-008-0489-2 PMC2522299 18322684

[121] Zhang J Yu J Yang L Li H Wei F Zhao H. Enhanced activation of human dendritic cells by silencing SOCS1 and activating TLRs simultaneously. Cancer Immunol Immunother (2012) 61(10):1653–61. doi: 10.1007/s00262-012-1218-4 PMC11028872 22366886

[122] McNutt M. Cancer immunotherapy. Science (2013) 342(6165):1417. doi: 10.1126/science.1249481 24357273

[123] Kamath SD Kalyan A Kircher S Nimeiri H Fought AJ Benson A. Ipilimumab and gemcitabine for advanced pancreatic cancer: A phase ib study. Oncologist (2020) 25(5):e808–15. doi: 10.1634/theoncologist.2019-0473 PMC7216436 31740568

[124] Weiss GJ Blaydorn L Beck J Bornemann-Kolatzki K Urnovitz H Schütz E. Phase Ib/II study of gemcitabine, nab-paclitaxel, and pembrolizumab in metastatic pancreatic adenocarcinoma. Invest New Drugs (2018) 36(1):96–102. doi: 10.1007/s10637-017-0525-1 29119276

[125] Aglietta M Barone C Sawyer MB Moore MJ Miller WH Jr Bagalà C. A phase I dose escalation trial of tremelimumab (CP-675,206) in combination with gemcitabine in chemotherapy-naive patients with metastatic pancreatic cancer. Ann Oncol (2014) 25(9):1750–5. doi: 10.1093/annonc/mdu205 24907635

[126] Sharma P Sohn J Shin SJ Oh DY Keam B Lee HJ. Efficacy and tolerability of tremelimumab in locally advanced or metastatic urothelial carcinoma patients who have failed first-line platinum-based chemotherapy. Clin Cancer Res (2020) 26(1):61–70. doi: 10.1158/1078-0432.CCR-19-1635 31801732

[127] Melisi D Garcia-Carbonero R Macarulla T Pezet D Deplanque G Fuchs M. Galunisertib plus gemcitabine vs. gemcitabine for first-line treatment of patients with unresectable pancreatic cancer. Br J Cancer (2018) 119(10):1208–14. doi: 10.1038/s41416-018-0246-z PMC6251034 30318515

[128] Melisi D Oh DY Hollebecque A Calvo E Varghese A Borazanci E. Safety and activity of the TGFβ receptor I kinase inhibitor galunisertib plus the anti-PD-L1 antibody durvalumab in metastatic pancreatic cancer. J Immunother Cancer (2021) 9(3):e002068. doi: 10.1136/jitc-2020-002068 33688022 PMC7944986

[129] Provenzano PP Cuevas C Chang AE Goel VK Von Hoff DD Hingorani SR. Enzymatic targeting of the stroma ablates physical barriers to treatment of pancreatic ductal adenocarcinoma. Cancer Cell (2012) 21(3):418–29. doi: 10.1016/j.ccr.2012.01.007 PMC3371414 22439937

[130] Ramanathan RK McDonough SL Philip PA Hingorani SR Lacy J Kortmansky JS. Phase IB/II randomized study of FOLFIRINOX plus pegylated recombinant human hyaluronidase versus FOLFIRINOX alone in patients with metastatic pancreatic adenocarcinoma: SWOG S1313. J Clin Oncol (2019) 37(13):1062–9. doi: 10.1200/JCO.18.01295 PMC6494359 30817250

[131] Hingorani SR Harris WP Beck JT Berdov BA Wagner SA Pshevlotsky EM. Phase ib study of PEGylated recombinant human hyaluronidase and gemcitabine in patients with advanced pancreatic cancer. Clin Cancer Res (2016) 22(12):2848–54. doi: 10.1158/1078-0432.CCR-15-2010 PMC7787348 26813359

[132] Overman M Javle M Davis RE Vats P Kumar-Sinha C Xiao L. Randomized phase II study of the bruton tyrosine kinase inhibitor acalabrutinib, alone or with pembrolizumab in patients with advanced pancreatic cancer. J Immunother Cancer (2020) 8(1):e000587. doi: 10.1136/jitc-2020-000587 32114502 PMC7057435

[133] Karakhanova S Mosl B Harig S von Ahn K Fritz J Schmidt J. Influence of interferon-alpha combined with chemo (radio) therapy on immunological parameters in pancreatic adenocarcinoma. Int J Mol Sci (2014) 15(3):4104–25. doi: 10.3390/ijms15034104 PMC3975387 24608924

[134] Scheid C Young R McDermott R Fitzsimmons L Scarffe JH Stern PL. Immune function of patients receiving recombinant human interleukin-6 (IL-6) in a phase I clinical study: induction of c-reactive protein and IgE and inhibition of natural killer and lymphokine-activated killer cell activity. Cancer Immunol Immunother (1994) 38(2):119–26. doi: 10.1007/BF01526207 PMC11038782 8306367

[135] Bockorny B Semenisty V Macarulla T Borazanci E Wolpin BM Stemmer SM. BL-8040, a CXCR4 antagonist, in combination with pembrolizumab and chemotherapy for pancreatic cancer: the COMBAT trial. Nat Med (2020) 26(6):878–85. doi: 10.1038/s41591-020-0880-x 32451495

[136] Rong Y Qin X Jin D Lou W Wu L Wang D. A phase I pilot trial of MUC1-peptide-pulsed dendritic cells in the treatment of advanced pancreatic cancer. Clin Exp Med (2012) 12(3):173–80. doi: 10.1007/s10238-011-0159-0 21932124

[137] Kondo H Hazama S Kawaoka T Yoshino S Yoshida S Tokuno K. Adoptive immunotherapy for pancreatic cancer using MUC1 peptide-pulsed dendritic cells and activated T lymphocytes. Anticancer Res (2008) 28(1B):379–87. 18383873

[138] Lepisto AJ Moser AJ Zeh H Lee K Bartlett D McKolanis JR. A phase I/II study of a MUC1 peptide pulsed autologous dendritic cell vaccine as adjuvant therapy in patients with resected pancreatic and biliary tumors. Cancer Ther (2008) 6(B):955–64. PMC2614325 19129927

[139] Koido S Homma S Okamoto M Takakura K Mori M Yoshizaki S. Treatment with chemotherapy and dendritic cells pulsed with multiple wilms' tumor 1 (WT1)-specific MHC class I/II-restricted epitopes for pancreatic cancer. Clin Cancer Res (2014) 20(16):4228–39. doi: 10.1158/1078-0432.CCR-14-0314 25056373

[140] Mehrotra S Britten CD Chin S Garrett-Mayer E Cloud CA Li M. Vaccination with poly(IC:LC) and peptide-pulsed autologous dendritic cells in patients with pancreatic cancer. J Hematol Oncol (2017) 10(1):82. doi: 10.1186/s13045-017-0459-2 28388966 PMC5384142

[141] Lin M Liang S Wang X Liang Y Zhang M Chen J. Short-term clinical efficacy of percutaneous irreversible electroporation combined with allogeneic natural killer cell for treating metastatic pancreatic cancer. Immunol Lett (2017) 186:20–7. doi: 10.1016/j.imlet.2017.03.018 28392199

[142] Lin M Liang S Wang X Liang Y Zhang M Chen J. Percutaneous irreversible electroporation combined with allogeneic natural killer cell immunotherapy for patients with unresectable (stage III/IV) pancreatic cancer: a promising treatment. J Cancer Res Clin Oncol (2017) 143(12):2607–18. doi: 10.1007/s00432-017-2513-4 PMC11819031 28871458

[143] Ullenhag GJ Mozaffari F Broberg M Mellstedt H Liljefors M. Clinical and immune effects of lenalidomide in combination with gemcitabine in patients with advanced pancreatic cancer. PloS One (2017) 12(1):e0169736. doi: 10.1371/journal.pone.0169736 28099502 PMC5242484

[144] McCracken MN Cha AC Weissman IL. Molecular pathways: Activating T cells after cancer cell phagocytosis from blockade of CD47 "Don't eat me" signals. Clin Cancer Res (2015) 21(16):3597–601. doi: 10.1158/1078-0432.CCR-14-2520 PMC4621226 26116271

[145] Kamerkar S LeBleu VS Sugimoto H Yang S Ruivo CF Melo SA. Exosomes facilitate therapeutic targeting of oncogenic KRAS in pancreatic cancer. Nature (2017) 546(7659):498–503. doi: 10.1038/nature22341 28607485 PMC5538883

[146] Koh E Lee EJ Nam GH Hong Y Cho E Yang Y. Exosome-SIRPα, a CD47 blockade increases cancer cell phagocytosis. Biomaterials (2017) 121:121–9. doi: 10.1016/j.biomaterials.2017.01.004 28086180

[147] Morishita M Takahashi Y Matsumoto A Nishikawa M Takakura Y. Exosome-based tumor antigens-adjuvant co-delivery utilizing genetically engineered tumor cell-derived exosomes with immunostimulatory CpG DNA. Biomaterials (2016) 111:55–65. doi: 10.1016/j.biomaterials.2016.09.031 27723556

[148] Xie Y Bai O Zhang H Yuan J Zong S Chibbar R. Membrane-bound HSP70-engineered myeloma cell-derived exosomes stimulate more efficient CD8(+) CTL- and NK-mediated antitumour immunity than exosomes released from heat-shocked tumour cells expressing cytoplasmic HSP70. J Cell Mol Med (2010) 14(11):2655–66. doi: 10.1111/j.1582-4934.2009.00851.x PMC4373481 19627400

[149] Hao S Bai O Yuan J Qureshi M Xiang J. Dendritic cell-derived exosomes stimulate stronger CD8+ CTL responses and antitumor immunity than tumor cell-derived exosomes. Cell Mol Immunol (2006) 3(3):205–11. 16893501

[150] Morse MA Garst J Osada T Khan S Hobeika A Clay TM. A phase I study of dexosome immunotherapy in patients with advanced non-small cell lung cancer. J Transl Med (2005) 3(1):9. doi: 10.1186/1479-5876-3-9 15723705 PMC551593

[151] Seo N Shirakura Y Tahara Y Momose F Harada N Ikeda H. Activated CD8+ T cell extracellular vesicles prevent tumour progression by targeting of lesional mesenchymal cells. Nat Commun (2018) 9(1):435. doi: 10.1038/s41467-018-02865-1 29382847 PMC5789986

[152] Lugini L Cecchetti S Huber V Luciani F Macchia G Spadaro F. Immune surveillance properties of human NK cell-derived exosomes. J Immunol (2012) 189(6):2833–42. doi: 10.4049/jimmunol.1101988 22904309

[153] Cheng L Wang Y Huang L. Exosomes from M1-polarized macrophages potentiate the cancer vaccine by creating a pro-inflammatory microenvironment in the lymph node. Mol Ther (2017) 25(7):1665–75. doi: 10.1016/j.ymthe.2017.02.007 PMC5498801 28284981

[154] Morrison AH Byrne KT Vonderheide RH. Immunotherapy and prevention of pancreatic cancer. Trends Cancer (2018) 4(6):418–28. doi: 10.1016/j.trecan.2018.04.001 PMC6028935 29860986

[155] AstraZeneca. In: Study of tremelimumab in patients with advanced solid tumors. Available at: https://clinicaltrials.gov/show/NCT02527434.

[156] Varricchi G Galdiero MR Marone G Criscuolo G Triassi M Bonaduce D. Cardiotoxicity of immune checkpoint inhibitors. ESMO Open (2017) 2(4):e000247. doi: 10.1136/esmoopen-2017-000247 29104763 PMC5663252

[157] Hosein AN Brekken RA Maitra A. Pancreatic cancer stroma: an update on therapeutic targeting strategies. Nat Rev Gastroenterol Hepatol (2020) 17(8):487–505. doi: 10.1038/s41575-020-0300-1 32393771 PMC8284850

[158] Chen J Ding ZY Li S Liu S Xiao C Li Z. Targeting transforming growth factor-β signaling for enhanced cancer chemotherapy. Theranostics (2021) 11(3):1345–63. doi: 10.7150/thno.51383 PMC7738904 33391538

[159] Gunderson AJ Kaneda MM Tsujikawa T Nguyen AV Affara NI Ruffell B. Bruton tyrosine kinase-dependent immune cell cross-talk drives pancreas cancer. Cancer Discovery (2016) 6(3):270–85. doi: 10.1158/2159-8290.CD-15-0827 PMC4783268 26715645

[160] Öhlund D Handly-Santana A Biffi G Elyada E Almeida AS Ponz-Sarvise M. Distinct populations of inflammatory fibroblasts and myofibroblasts in pancreatic cancer. J Exp Med (2017) 214(3):579–96. doi: 10.1084/jem.20162024 PMC5339682 28232471

[161] Stift A Friedl J Dubsky P Bachleitner-Hofmann T Schueller G Zontsich T. Dendritic cell-based vaccination in solid cancer. J Clin Oncol (2003) 21(1):135–42. doi: 10.1200/JCO.2003.02.135 12506182

[162] Nakamura M Wada J Suzuki H Tanaka M Katano M Morisaki T. Long-term outcome of immunotherapy for patients with refractory pancreatic cancer. Anticancer Res (2009) 29(3):831–6. 19414316

[163] Alahdal M Xing Y Tang T Liang J. 1-Methyl-D-tryptophan reduces tumor CD133+ cells, wnt/β-catenin and NF-κβp65 while enhances lymphocytes NF-κβ2, STAT3, and STAT4 pathways in murine pancreatic adenocarcinoma. Sci Rep (2018) 8(1):9869. doi: 10.1038/s41598-018-28238-8 29959375 PMC6026162

[164] Li Y Zhao W Wang Y Wang H Liu S. Extracellular vesicle-mediated crosstalk between pancreatic cancer and stromal cells in the tumor microenvironment. J Nanobiotechnol (2022) 20(1):208. doi: 10.1186/s12951-022-01382-0 PMC9063273 35501802

[165] Moeng S Son SW Lee JS Lee HY Kim TH Choi SY. Extracellular vesicles (EVs) and pancreatic cancer: From the role of EVs to the interference with EV-mediated reciprocal communication. Biomedicines (2020) 8(8):267. doi: 10.3390/biomedicines8080267 32756339 PMC7459718

[166] Nannan L Oudart JB Monboisse JC Ramont L Brassart-Pasco S Brassart B. Extracellular vesicle-dependent cross-talk in cancer-focus on pancreatic cancer. Front Oncol (2020) 10:1456. doi: 10.3389/fonc.2020.01456 32974169 PMC7466446

[167] Zhao J Schlößer HA Wang Z Qin J Li J Popp F. Tumor-derived extracellular vesicles inhibit natural killer cell function in pancreatic cancer. Cancers (Basel) (2019) 11(6):874. doi: 10.3390/cancers11060874 31234517 PMC6628179

[168] Xie F Zhou X Fang M Li H Su P Tu Y. Extracellular vesicles in cancer immune microenvironment and cancer immunotherapy. Adv Sci (Weinh) (2019) 6(24):1901779. doi: 10.1002/advs.201901779 31871860 PMC6918121

[169] Javadrashid D Baghbanzadeh A Derakhshani A Leone P Silvestris N Racanelli V. Pancreatic cancer signaling pathways, genetic alterations, and tumor microenvironment: The barriers affecting the method of treatment. Biomedicines (2021) 9(4):373. doi: 10.3390/biomedicines9040373 33918146 PMC8067185

[170] Qian Y Gong Y Fan Z Luo G Huang Q Deng S. Molecular alterations and targeted therapy in pancreatic ductal adenocarcinoma. J Hematol Oncol (2020) 13(1):130. doi: 10.1186/s13045-020-00958-3 33008426 PMC7532113

[171] Alagesan B Contino G Guimaraes AR Corcoran RB Deshpande V Wojtkiewicz GR. Combined MEK and PI3K inhibition in a mouse model of pancreatic cancer. Clin Cancer Res (2015) 21(2):396–404. doi: 10.1158/1078-0432.CCR-14-1591 25348516 PMC4447091

[172] Smalley I Smalley KSM. ERK inhibition: A new front in the war against MAPK pathway-driven cancers? Cancer Discovery (2018) 8(2):140–2. doi: 10.1158/2159-8290.CD-17-1355 29431672

[173] Bear AS Vonderheide RH O'Hara MH. Challenges and opportunities for pancreatic cancer immunotherapy. Cancer Cell (2020) 38(6):788–802. doi: 10.1016/j.ccell.2020.08.004 32946773 PMC7738380

[174] Rocha FG. Landmark series: Immunotherapy and targeted therapy for pancreatic cancer. Ann Surg Oncol (2021) 28(3):1400–6. doi: 10.1245/s10434-020-09367-9 33386541

[175] Balachandran VP Beatty GL Dougan SK. Broadening the impact of immunotherapy to pancreatic cancer: Challenges and opportunities. Gastroenterology (2019) 156(7):2056–72. doi: 10.1053/j.gastro.2018.12.038 PMC6486864 30660727

[176] Schizas D Charalampakis N Kole C Economopoulou P Koustas E Gkotsis E. Immunotherapy for pancreatic cancer: A 2020 update. Cancer Treat Rev (2020) 86:102016. doi: 10.1016/j.ctrv.2020.102016 32247999

